# The Roles of Consonant, Rime, and Tone in Mandarin Spoken Word Recognition: An Eye-Tracking Study

**DOI:** 10.3389/fpsyg.2021.740444

**Published:** 2022-01-05

**Authors:** Ting Zou, Yutong Liu, Huiting Zhong

**Affiliations:** School of English and International Studies, Beijing Foreign Studies University, Beijing, China

**Keywords:** spoken word recognition, Mandarin, consonant, rime, tone

## Abstract

This study investigated the relative role of sub-syllabic components (initial consonant, rime, and tone) in spoken word recognition of Mandarin Chinese using an eye-tracking experiment with a visual world paradigm. Native Mandarin speakers (all born and grew up in Beijing) were presented with four pictures and an auditory stimulus. They were required to click the picture according to the sound stimulus they heard, and their eye movements were tracked during this process. For a target word (e.g., *tang2* “candy”), nine conditions of competitors were constructed in terms of the amount of their phonological overlap with the target: consonant competitor (e.g., *ti1* “ladder”), rime competitor (e.g., *lang4* “wave”), tone competitor (e.g., *niu2* “cow”), consonant plus rime competitor (e.g., *tang1*”soup”), consonant plus tone competitor (e.g., *tou2* “head”), rime plus tone competitor (e.g., *yang2* “sheep”), cohort competitor (e.g., *ta3* “tower”), cohort plus tone competitor (e.g., *tao2* “peach”), and baseline competitor (e.g., *xue3* “snow”). A growth curve analysis was conducted with the fixation to competitors, targets, and distractors, and the results showed that (1) competitors with consonant or rime overlap can be adequately activated, while tone overlap plays a weaker role since additional tonal information can strengthen the competitive effect only when it was added to a candidate that already bears much phonological similarity with the target. (2) Mandarin words are processed in an incremental way in the time course of word recognition since different partially overlapping competitors could be activated immediately; (3) like the pattern found in English, both cohort and rime competitors were activated to compete for lexical activation, but these two competitors were not temporally distinctive and mainly differed in the size of their competitive effects. Generally, the gradation of activation based on the phonological similarity between target and candidates found in this study was in line with the continuous mapping models and may reflect a strategy of native speakers shaped by the informative characteristics of the interaction among different sub-syllabic components.

## Introduction

Spoken word recognition forms the basis of auditory language comprehension. As the studies investigating real-time spoken word recognition accumulate, it is becoming increasingly clear that with the unfolding of the input, multiple words can be activated in parallel to competing during the recognition process (Frauenfelder and Tyler, 1987). Different models (such as the TRACE model: McClelland and Elman, 1986 and the Shortlist model: Norris, 1994) have been proposed to account for this dynamic process. However, since most of these models were developed with Indo-European languages, word-level prosody, such as lexical tones were not encoded.

As a typical tone language, Mandarin is significantly different from Indo-European languages, such as English, in the use of lexical tones and its morpheme-syllable mapping. Phonotactically, a syllable in Mandarin can be divided into three sub-syllabic components: initial consonant, rime, and tone. In the past decades, there has been a growing interest in examining tonal processing, but the question of whether the tone plays a weaker role compared to segments in Mandarin word recognition has received conflicting answers (e.g., Cutler and Chen, 1997; Schirmer et al., 2005; Malins and Joanisse, 2010; Sereno and Lee, 2015). The relative importance of onset and rime in Mandarin spoken word recognition still needs further investigation (Zhao et al., 2011; Malins and Joanisse, 2012a; Zou, 2017). Accounts on the nature of Mandarin word processing are also inconclusive, with some studies supporting an incremental fashion (e.g., Malins and Joanisse, 2012a; Ho et al., 2019), while others suggesting a holistic way of processing and emphasizing the special status of syllables (e.g., Zhao et al., 2011; Gao et al., 2019).

Therefore, this study was designed to revisit spoken word recognition in Mandarin Chinese. The goal was to bring in new data on monosyllabic word processing in order to test the relative contribution of sub-syllabic items, examine the weighting of cohort and rime in lexical access, as well as adjudicate the existing contradictory views on the incremental versus holistic processing of monosyllabic words. In doing so, we also aimed to reveal the general pattern of word processing in a tone language like Mandarin, a topic that has ramifications for theories and models of speech representation and recognition.

### The Role of Sub-Syllabic Components in Mandarin Spoken Word Recognition

As a typical tone language, Mandarin Chinese presents some distinct features in terms of syllable structure compared to Indo-European languages (such as English). First, apart from segmental information (i.e., consonants and vowels), Mandarin Chinese also incorporates vocal pitch information (i.e., lexical tones) to distinguish lexical meaning. For non-tone language speakers, pitch information is mainly used at the post-lexical level, such as marking pragmatic nuances and sentence modes, as well as signaling the grouping of words into higher units (see, e.g., Shattuck-Hufnagel and Turk, 1996; Cutler et al., 1997; Dahan and Gaskell, 2007 a detailed review). Mandarin Chinese speakers, on the other hand, primarily employ pitch configurations to differentiate between word forms. For example, the meaning of the syllable /ma/ can be “mother,” “hemp,” “horse,” and “to scold” when it is associated with Tone 1 (high-level pitch), Tone 2 (mid-rising pitch), Tone 3 (low-dipping pitch), and Tone 4 (high-falling pitch), respectively (Howie, 1974; Duanmu, 2000; Chen and Gussenhoven, 2008). The second significant feature is that the majority of morphemes are monosyllabic. Such morpheme-syllable mapping makes syllable an essential meaning-bearing unit, and therefore it has been suggested that syllable may merit a special status beyond and above phonemes in word processing (e.g., Mok, 2009; Zhao et al., 2011; Chen and Chen, 2013). Furthermore, Mandarin Chinese has a relatively simple interior syllable structure: a syllable in Mandarin generally includes a single-consonant as onset (optional), a vowel/diphthong as a rime and an optional coda (either an alveolar nasal /n/ or velar nasal /ŋ/) (cf. Duanmu, 2007). The number of possible syllable structures is therefore considerably smaller than that of some Indo-European languages (such as English). All these properties make monosyllabic words ideal for the investigation of spoken word recognition in Mandarin Chinese.

Prior research on Mandarin spoken word recognition mainly focuses on the relative contribution of segments and tones. With various tasks recording end-stage responses, early behavioral studies generally suggested that tones might be accessed later and make a less contribution in lexical access compared to segments (Repp and Lin, 1990; Taft and Chen, 1992; Cutler and Chen, 1997; Ye and Connie, 1999, Experiment 1; Yip, 2007). For instance, Repp and Lin (1990) found that in a speeded classification task, the tone was accessed later than segment information, as Mandarin speakers took longer to respond to tonal distinctions than both consonants and vowels. A few recent research also has obtained similar results (Tong et al., 2008; Hu et al., 2012; Sereno and Lee, 2015; Connell et al., 2016; Davis et al., 2016; Wiener and Turnbull, 2016).

This line of studies further suggested that the tone disadvantage may be in part due to the difference in temporal availability of the cues: tonal information becomes available only until enough part of its carrier, i.e., vowels, get processed. Another potential factor is the lower information value of tone suggested in Tong et al. (2008). According to the information-theoretic methods (Garner and Miller, 1988; Tong et al., 2008), the concept of information refers to what has happened relative to what could have happened but did not. By excluding the alternative options, the information reduces the uncertainty of an event. The larger the amount of information value is, the more alternative options are excluded, and thus less uncertain the event would be. In the context of word recognition, the ability of a given signal to constrain recognition is related to its probability of occurring in a corresponding communication system. Signals with a lower probability of occurrence have a greater ability to exclude alternative candidates and thus enjoy larger information value. Due to the small tone inventory compared to that of the segments, each tone occurs more frequently and associates with more words in Mandarin Chinese than consonants and rimes, which makes tone less informative and poorer at constraining word recognition.

More recent studies, on the other hand, have adopted online measures such as eye-tracking and event-related brain potentials (ERPs), and suggested that the mismatch in tone can constrain lexical access effectively like segments (Schirmer et al., 2005; Malins and Joanisse, 2010, 2012a; Malins et al., 2014). In an eye-tracking study (Malins and Joanisse, 2010) with a visual world paradigm, participants were asked to click the corresponding picture according to the auditory stimulus presented to them. The results demonstrated that, for a target such as *chuang2* “bed,” both the phonological competitor overlapping the target in all segments (*chuang1* “window”) and phonological competitor overlapping the target in consonant and initial part of rime (*chuan2* “boat”) distract fixations from the target to a similar degree, indicating that tones and phonemes are accessed concurrently and play comparable roles in constraining lexical activation. A subsequent ERP study (Malins and Joanisse, 2012a) confirmed that just like the use of segments, tonal information can be used to constrain word recognition as soon as it becomes available, although a less persistent and more left-lateralized effect in the tone-mismatch condition effect compared to the segment-mismatch condition indicated different mechanisms underlying the access to tonal versus phonemic information.

As mentioned before, since there is a clear syllable-morpheme mapping and the relatively simple interior syllable structure in Mandarin, the functional significance of the three sub-syllabic components (consonant, rime, and tone) and their real-time interaction could be of key importance in monosyllabic word recognition. A few studies (e.g., Tong et al., 2008; Wiener and Turnbull, 2016; Ling and Grüter, 2020; Shaw and Tyler, 2020) have tested the weighting of the three components. Some of them suggested a more important status of segments than tones, and within the segmental level, vowels (rime) seemed to play a more primary role than consonants (Tong et al., 2008; Wiener and Turnbull, 2016). For example, in a study by Wiener and Turnbull (2016), native speakers were asked to change non-words (e.g., *su3*) into words by manipulating a single consonant (e.g., *tu3*), vowel (e.g., *si3*), or tone (e.g., *su4*) in a word reconstruction task. The results suggested that Mandarin speakers rely more on vowels than consonants and tones in lexical access since changes to vowels were the slowest and least accurate.

Taken together, it could be the case that tones may need more time to develop than segments, thus, the tone-matching words are activated to a less extent compared to segment-matching candidates. However, concerning the effect of constraining lexical activation, both segments and tones could be accessed and used as soon as they become available and this effect can be captured and revealed more effectively in online measurements. Within the segmental level, vowels (rime) seemed to play a more primary role than consonants. But the specific roles of consonant, rime, and tone, as well as their interaction in the time course of spoken word recognition have not been investigated using on-line tasks. In the current study, these issues were tested in an eye-tracking experiment with a visual world paradigm. Moreover, since Tong et al. (2008) suggested that information value could account for the relative role of tones and segments in word processing, the informativeness of individual sub-syllabic components and their combinations was also estimated in the current study to investigate the potential link between a component’s information value and its contribution in lexical activation.

### Incremental Versus Holistic Processing in Mandarin Spoken Word Processing

In addition to the issue of the role of sub-syllabic components in word processing, whether Mandarin monosyllabic words are processed in an incremental way or in a more holistic fashion and which current model(s) would be optimal to account for lexical activation in Mandarin have also attracted much research attention.

Among the mixed findings obtained from both behavior and neuropsychological studies, some suggested that Mandarin monosyllabic words are processed in an incremental fashion and syllable does not have a special status. For example, in Malins and Joanisse (2012a), ERPs were recorded while participants performed an auditory word-picture matching task in which auditory words and picture names shared different degrees of phonological similarity. The rhyme, tonal, and unrelated conditions all exhibited similar effects in PMN and N400, indicating that the whole-syllable mismatches do not merit any special status beyond and above individual sub-syllabic components. A recent study focused on spoken word processing in sentence context also suggested that Mandarin syllables are processed incrementally through phonological segments (Ho et al., 2019). As indicated by the authors, online processing models (e.g., TRACE) are suitable to account for the incremental processing pattern of Mandarin monosyllabic words. This type of online processing models such as TRACE (McClelland and Elman, 1986) and Cohort (Marslen-Wilson, 1987) assume that lexical candidates are activated immediately with receipt of a minimal amount of acoustic information. Speech sounds presented at a feature layer can be mapped onto phoneme and word layers. With between-layer excitation and within-layer competitive inhibition, the activation is updated incrementally, and multiple words are activated in parallel to compete with the target word being recognized. Several attempts have been made to accommodate Mandarin spoken word processing to the TRACE model. Ye and Connie (1999) first extended the TRACE model by adding a separate level of “toneme node” in contrast to the “phoneme node.” The model assigns tone and segment to separate representation, and the feedback from lexicon to toneme is considered to be stronger than the phoneme-lexicon connection. This concept of “toneme” has been adopted in almost all later modifications to the TRACE model. For instance, the TRACE-T model proposed by Malins and Joanisse (2012b) specified the acoustic feature layer for both segment and tone. Different from the above modified versions of the TRACE model suggesting potential independent processing for phonemes and tonemes, the TTRACE model proposed by Tong et al. (2014) postulated an integral representation of tonemes and phonemes based on the empirical findings of strong integral processing of vowel and tone (Tong et al., 2008, 2014). More specifically, it specified the correlation of activation magnitude and the degree of segmental and suprasegmental overlap among words. For the target word /fu1/ (“skin”), there can be distinct levels of activation for the competitive candidates. /wu1/ (“black,” sharing the same vowel and tone), /fu6/ (“father,” sharing the same consonant and vowel), and /fa1/ (“flower,” sharing the same consonant and tone) can be activated to the strongest degree since they share two of the three aspects with the target. While /ku2/ (“ancient,” sharing the same vowel only), /fɔ2/ (“fire,” sharing the same consonant only), and /tɔ1/ (“many” sharing the same tone only) may be activated to a smaller degree since they share only one aspect with the target. In a study by Choi et al. (2017), tone-mismatch negativity (MMN), vowel-MMN, and double-MMN were elicited in a passive oddball paradigm. The results showed that double-MMN were significantly smaller in amplitude than the sum of single feature MMNs. Based on this perceptual vowel-tone integration, the TTRACE + model was proposed in which vowels and tones are encoded in the same memory trace and treated as the same phonological unit.

On the other hand, the neighborhood activation model (NAM; Luce and Pisoni, 1998) posited that words are activated based on their global similarity to the input signal. The set of words that can be activated as candidates are “neighbors” of the target word: words that maximally differ with the input by one phoneme in any position. Since NAM emphasizes the global similarity and neglects the temporal difference of the competitors, both onset and rhyme competitors will generate equal influences on the activation of the target words.

There is also evidence suggesting that syllable may play a special role in word recognition, and the processing of Mandarin words can be accounted for in a more holistic way as depicted in NAM (e.g., Zhou and Marslen-Wilson, 1994; Zhao et al., 2011; Sereno and Lee, 2015). For example, an ERP study by Zhao et al. (2011) explored monosyllabic Mandarin word processing using a picture matching task, in which the participants were asked to judge whether a target picture and a subsequent picture belonged to the same semantic category with a spoken word presented between the displays of the two pictures as a phonological competitor. The ERPs recorded during the processing of the auditory words revealed that the whole-syllable mismatching words (target picture: *bi2*, syllable mismatching word: *ge1*) elicited a considerable earlier and stronger N400 than the partial mismatched words (target picture: *bi2*; onset mismatch: *li2*, rime mismatch: *bo2*, and tone mismatch: *bi3*), indicating that the whole syllable may merit a special status over phoneme and tone in lexical recognition. Moreover, equivalent N400 effects were found in both amplitudes and time course for onset (e.g., *li2*), rime (e.g., *bo2*), and tone (e.g., *bi3*) mismatching words, which is also in line with NAM, since this model emphasizes the global mapping and does not predict temporally distinct effects for different partial violations. These findings are reminiscent of other studies treating syllables as an essential unit for perceiving and producing Mandarin words (Chen et al., 2002; Mok, 2009; O’Seaghdha et al., 2010; You et al., 2012; Chen and Chen, 2013).

### The Weighting of Cohorts and Rimes in Mandarin Monosyllabic Word Processing

Among the online word-processing models, the distinction of the feedforward Cohort model and the continuous mapping TRACE model lies in the role of cohort competitors (sharing word-initial part with the target) and rime competitors (sharing word-final part with the target). The Cohort model highlights the weight of the onset of a syllable during word recognition, predicting the words diverging from the onset with the input will not be accessed, while TRACE emphasizes continuous mapping and allows for the competition from rhyme-overlapping words, but predicts a weaker rhyme effect compared to onset effect. For example, as exhibited in the evidence supporting TRACE (Allopenna et al., 1998), both cohort competitor (e.g., *beetle*) and rhyme competitor (e.g., *speaker*) could be activated for the target word (e.g., *beaker*), but *speaker* was activated later and to a smaller extent.

Previous findings in English word processing are generally in line with the TRACE model, suggesting that both competitors showed a clear competitive effect, but the effect is earlier and stronger for cohort competitors (e.g., target: *cake*, competitor: *cage*) than rime competitors (e.g., target: *cake*, competitor: *rake*) (Allopenna et al., 1998; Desroches et al., 2009). Past studies on Mandarin word processing, however, have documented divergent results. In an eye-tracking study by Malins and Joanisse (2010), the rhyming words failed to generate a competitive effect, but a later ERP study (Zhao et al., 2011) demonstrated the opposite, suggesting that rimes play a stronger role in Mandarin compared to that in English, equivalent to the role of onsets, and there is no temporal distinction between them. A stronger role of rime and tone overlap over initial consonant and tone overlap has also been found in an eye-tracking study (Zou, 2017; Zou and Chen, 2019). In another ERP study (Malins and Joanisse, 2012a), a similar pattern between Mandarin and English in the weighting of onsets and rimes was demonstrated.

Given the state-of-art, these mixed results on the processing pattern of Mandarin spoken words were attained from studies using various experimental paradigms and different conditions of phonological similarity among words. More systematic studies are clearly needed to capture the dynamics in the time course of Mandarin word processing and measure the contribution of different sub-syllabic components. Moreover, whether Mandarin words are processed incrementally or in a more holistic fashion, as well as the relative contributions of cohort and rime overlaps also call for a more fine-grained investigation, so that we can verify the basic pattern of word processing before fruitful discussions become possible on the modeling of the recognition process.

### Rationale for the Current Study

Using a visual world paradigm, the current study sought to examine (1) the specific roles of consonant, rime, tone, as well as their combinations in the process of monosyllabic Mandarin word recognition, (2) whether monosyllabic Mandarin words are processed in an incremental way or in a more holistic manner, and (3) whether cohort and rime competitors play a similar role as predicted by TRACE model. The results were also discussed with respect to the modification of existing models in order to accommodate tones.

Visual world paradigm (VWP) has typically been used in eye-tracking studies to investigate online auditory word recognition (Tanenhaus et al., 1995; also see a review in Huettig et al., 2011). In a VWP task, participants are presented with a display of four pictures and an auditory stimulus corresponding to one of these pictures, and they are asked to identify the word (i.e., the target) they heard with their eye movements being tracked. The target word is always presented with a phonologically similar competitor and two phonologically unrelated distractors. Clear evidence has been found in support of a linking hypothesis between activation levels and the probability of fixations launched to the corresponding picture (Allopenna et al., 1998), which indicates that eye-movement measures are well-suited to examining how fine-grained acoustic information affect word recognition. Since it can tap directly into the time course of word processing, the eye-tracking method has also been adopted to reveal the fine-grained details in how stress information was evaluated online and used to speed up spoken word recognition by English speakers (Reinisch et al., 2010; Jesse et al., 2017).

To take a close examination of the role of individual sub-syllabic components, it is of key importance to use a relatively whole set of competitor conditions bearing various degrees of phonological similarity with the target, as suggested in Cutler and Chen (1997) and Yip (2007). Thus, nine conditions with different degrees of target-competitor similarity were included. Namely, a consonant (C) competitor sharing only consonant with the target (e.g., target word: *tang2* “candy,” competitor: *ti1* “ladder”), a rime (R) competitor sharing only rime (e.g., *lang4* “wave”), a tone (T) competitor sharing only tone (e.g., *niu2* “cow”), a consonant plus rime (CR) competitor sharing consonant and rime (e.g., *tang1* “soup”), a consonant plus tone condition (CT) (e.g., *tou2* “head”), a rime plus tone competitor (RT) (e.g., *yang2* “sheep”), and a baseline condition with a phonological non-competitor (B) (e.g., *xue3* “snow”). In addition, a cohort condition in which the competitor overlapped the target in the onset and the initial proportion of rime (COH) (e.g., *ta3* “tower”), as well as a cohort plus tone condition (COHT) (e.g., tao2 “peach”), were also incorporated.

In order to address the first issue, we compared the proportion of fixations to the C, R, and T competitors to unrelated distractors to reveal the competitive effect of competitors with overlap only in one component following the conventional method of visual world paradigm (Allopenna et al., 1998; Malins and Joanisse, 2010). Whether the competitors sharing two sub-syllabic components can be effectively activated was examined by comparing the proportion of fixations to competitor versus unrelated distractors in the CR, CT, and RT conditions. The relative contribution of consonant, rime, and tone was illustrated by cross competitor comparisons. Specifically, whether the additional consonant overlap would strengthen the competitive effect was shown by comparing the T versus CT conditions, as well as R versus CR conditions; the contribution of rime was tested by comparing T versus RT conditions and C versus CR conditions; and the contribution of tone, likewise, could be illustrated by comparing C versus CT and R versus RT conditions. To address the second research question, we took a close look at the magnitude and time course of activation for each competitor condition. If a monosyllabic word is processed in a holistic manner, then only neighbors differing from the target in one phoneme (i.e., the CR, RT, and COHT competitors) will be activated adequately, while the immediate activation of partially overlapping competitors will indicate an incremental way of processing. Lastly, with respect to the third issue, comparisons were made between COH versus R competitors as well as COHT versus RT competitors to show the relative weighting of onsets and rimes in word recognition.

## Materials and Methods

### Participants

Twenty-eight adult native Mandarin speakers (12 males and 16 females) were recruited to participate in this study. All of them were born and raised in Beijing and used standard Chinese in daily communication. They are undergraduate or graduate students from the Beijing Foreign Studies University, with an average age of 20.61 (SD = 2.45). None of them have any speech, hearing, or reading disorders. In addition, eight students were invited to rate the degree that the image stimuli match the intended Mandarin words used in the experiment. The participants were paid, and written consent was obtained from all of them.

### Stimuli

As shown in the Supplementary Material twelve sets of ten monosyllabic Mandarin words were selected as the stimuli for the eye-tracking experiment.

Previous studies on tonal discrimination suggested that some tone contrasts (e.g., Tone 2 versus Tone 3) might be harder to distinguish than other contrasts even for adult native Mandarin speakers (e.g., Bent, 2005; Zhang et al., 2012; Ma et al., 2017). To cancel out the discrepancy in processing difficulty across different tone pairs, all twelve possible tone contrasts were covered in the set of target words and their CR competitors. Moreover, we tried to avoid using words repeatedly across sets, but due to the limited number of picturable words, three of them were used twice (i.e., *guan4* “can,” *gai4* “lid,” *ti1* “ladder”), yielding a total of 117 monosyllabic Mandarin words used as stimuli. Another 24 Mandarin monosyllabic words were selected as stimuli for the practice session. In addition, all competitors were used as distractors for other sets to balance the occurrence of target and competitor words. In order to avoid the influence caused by word frequency, the Chinese Lexical Database (Sun et al., 2018) was used to compute the frequencies of the stimuli. One-way ANOVAs showed no significant differences between word frequencies across all tone-pair sets [*F* (11, 107) = 1.39, *p* > 0.05] and across conditions [*F* (9, 109) = 1.61, *p* > 0.05].

Although the number of picturable nouns which could meet our phonological requirements was limited, the effort was made to control the attractiveness of the stimuli. First, all the words were depicted with a black-and-white line drawing to achieve higher consistency in picture complexity. Second, in terms of the animacy of the referents, the number of animated words is comparable across conditions. For most competitor conditions, there are one or two animal words, and only T condition consists of four animal words (Detailed information is presented in the Supplementary Material). Moreover, before the analysis of eye-movement data, the fixation dwelling time for targets, competitors, and distractors in the preview section (before the presentation of the auditory stimuli) was inspected for each condition to reveal any potential preference due to the differential attractiveness of the referents (see sections “Data Analysis” and “Eye-Movement Data” for the method used and results for the preview analysis).

In addition, prior studies suggested that in Mandarin Chinese, the orthography similarity can influence spoken word recognition, since during spoken word processing, codes for written forms are also available, even if no written words are presented (Ziegler and Ferrand, 1998; Zou et al., 2012; Chen et al., 2016). That is, if the target and competitor words are phonograms sharing similar phonetic components at the written level, the results of activation would have been biased. Therefore, the stimuli in the present study have been checked to make sure that in all conditions, the targets and competitors do not share similar phonetic components in their written forms (see the Supplementary Material for the written forms of the words).

To ensure that the participants can immediately associate the picture with the intended word when they see it on the screen during the experiment, eight adult native Mandarin speakers were invited to rate the matching degree between the words and the corresponding pictures on a scale of one to seven, with seven points representing a perfect match. All pictures were rated higher than 5.50, and 113 out of 117 pictures got a rating point higher than 6, suggesting that they can precisely stand for the words. Averaged rating for the referent-picture matching is 6.69 for targets, 6.93 for C competitors, 6.83 for R competitors, 6.85 for T competitors, 6.66 for CR competitors, 6.70 for CT competitors, 6.88 for RT competitors, 6.69 for COH competitors, 6.68 for COHT competitors, and 6.91 for baseline condition. One-way ANOVA suggested no significant difference for the picture-word matching across conditions [*F* (8, 99) = 1.81, *p* > 0.05]. The rating results for individual words are provided in the Supplementary Material.

All the auditory words were recorded using the Elektroakustik M82 microphone (TELEFUNKEN Licences GmbH, Frankfurt, Germany) in a sound-attenuated lab at Beijing Foreign Studies University (44.1 kHz, 16 bit) by an adult female native Mandarin speaker (age = 21) from Beijing at a normal speech rate. The words were recalled three times in random order and two were selected as the final auditory stimuli. All auditory stimuli were then checked by a native Mandarin speaker to make sure that they can be correctly identified. The sound intensity was normalized to 73.3 dB, the average of all the sounds.

### Procedure

The participants were tested individually in the Artificial Intelligence and Human Languages Lab at Beijing Foreign Studies University. Before the eye-tracking experiment, they were asked to complete a self-paced familiarization task *via* E-Prime 3.0 software (Psychology Software Tools, Pittsburgh, PA, United States) to build the association of the word-picture pairs. Then, they were presented with a black-white line drawing at a time and were asked to speak out aloud the corresponding word. The number of times the participants produced an unexpected name was 6 for baseline condition, 7 for C competitors, 8 for R competitors, 8 for T competitors, 16 for CR competitors, 16 for CT competitors, 9 for RT competitors, 12 for COH competitors, and 22 for COHT competitors. The data for each word is presented in the Supplementary Material. When a participant produced an unexpected name for the picture, the intended name would be given and the picture would be tested again later. Therefore, although the total numbers of initial picture naming errors varied across conditions, all the pictures were correctly identified and named before the eye-tracking experiment.

**FIGURE 1:**
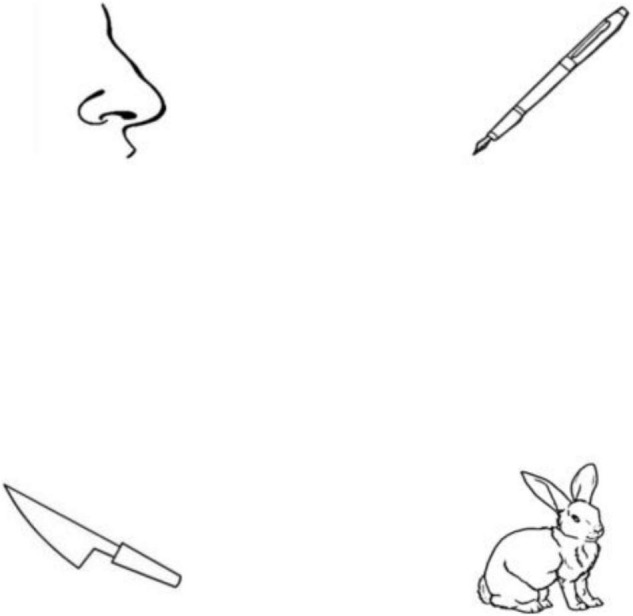
An example of visual stimuli display. This display consists of pictures of a target item (*bi3* “pen”), a phonological competitor item (*bi2* “nose”), and two phonologically unrelated distractor items (*tu4* “rabbit” and *dao1* “knife”).

In the eye-tracking experiments, participants performed an auditory signal-visual picture matching task in which eye movements were recorded by an Eyelink 1000 Plus (SR Research, Ottawa, ON, Canada) at a 500-Hz sampling rate with a 35 mm lens [This sampling rate is appropriate to track the eye movements for the visual world paradigm. Also see Malins and Joanisse (2010) and Huettig et al. (2011)]. The participants were seated in front of the screen with a chin rest set at a distance of 60 cm. In each trial, four images would be presented on the screen, corresponding to a target, a phonological competitor, and two phonologically unrelated distractors (e.g., Figure 1). To guarantee that the parafoveal view would not overlap during picture looking (Miellet et al., 2009; Li, 2016), the size and position of the visual stimuli were calculated according to the resolution (1,024 × 768 pixels) and the physical size (37.5 × 30.5 cm) of the display screen. The resolution of each picture in the current experiment was 161 × 149 pixels, subtending an eight-degree visual angle (Miellet et al., 2009). Before the experiment, participants were explained the task clearly and finished a nine-point calibration and validation. To get the participants familiar with the experimental procedure, a practice block consisting of twelve trials was implemented before the critical experimental blocks.

The task consisted of six blocks of 72 trials, i.e., 432 trials in total (12 trials × 9 conditions × 2 target-competitor relations × 2 repetitions). For half of the trials, the target-competitor relationship was presented as in the Supplementary Material, while, for the other half, the relationship was inverted to ensure that in all trials the probability for participants to hear the target or the competitor was the same. The positions of targets and competitors, i.e., upper-left, upper-right, lower-left, lower-right on the screen, and the relationship between their positions, i.e., opposite or adjacent, were counterbalanced across trials. Furthermore, the order of the trials for competitor conditions was randomized, and each target word, as well as competitor words within the same set, would not be heard adjacently.

**FIGURE 2:**
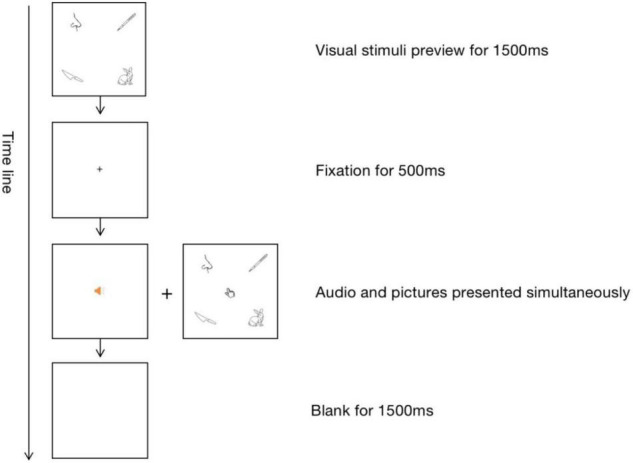
The procedure of the auditory-visual picture matching task.

The procedure of each trial was shown in Figure 2. First, the four-picture array was presented as a preview for 1,500 ms. Next, the pictures disappeared, followed by the appearance of the central fixation. Participants were asked to look at the fixation cross when it appeared. After 500 ms, the participants would hear an auditory word *via* headphones and simultaneously see the picture array again. They were instructed to select the picture corresponding to the auditory word by moving and clicking the mouse. Before the next trial started, there was a 1,500 ms blank as an interval. The whole experiment lasted for approximately an hour.

### Data Analysis

In each trial, the display screen contained four areas of interest corresponding to the target, the competitor, and two distractors. Only fixations located within these areas were coded as looks to the items. The data of reciprocal trials were left out and only the trials with the critical word as the target were analyzed. Additionally, trials in which participants blinked within the interest period from the audio onset to the mouse click (535 out of 12,096 trials) and the trials in which participants failed to click the target picture (1 out of 12,096 trials) were also excluded.

To examine whether any pictures were more attractive/attention-catching among the target, competitor, and distractors in each competitor condition, the fixation dwelling time in the four interest areas in the preview section was extracted. A one-way repeated-measure ANOVA was conducted for individual competitor conditions with the fixation dwelling time for each interest area as the dependent variable and the role of each interest area (i.e., target, competitor, and distractors) as the independent variable to test whether there were systematic biases due to picture attractiveness before the auditory stimulus was presented.

After the preview section, proportions of fixations were extracted from 200 ms after the onset of both visual and auditory stimuli (since it takes about 200 ms to launch an eye movement, cf. Hallet, 1986; Kamide et al., 2004) to 1,100 ms, the approximate position of the maximum proportion of looks to target (Malins and Joanisse, 2010). The data obtained at the sampling rate of 500 Hz were resampled at 62.5 Hz during analysis (Malins and Joanisse, 2010), and the subsequent plotting and modeling were based on the resampled data.

Growth curve analysis (GCA; Mirman et al., 2008; Mirman, 2014) was used to analyze the eye movement data with linear mixed modeling in R (Bates et al., 2014). The changing of the gaze distribution probability over time was captured and fitted using four-order orthogonal polynomials to reflect the minor changes of the eye-fixation patterns corresponding to different phonological manipulation. The intercept term represents the overall average height of the curve, the linear term indexes a monotonic change in the general direction of the curve, while the quadratic, cubic, and quartic terms tend to reflect the minor details of the steepness of the curves (Mirman et al., 2008).

For the fixation data, both curves of competitor look and target look were modeled with fourth-order orthogonal polynomials (linear, quadratic, cubic, and quartic components) and fixed effects of Condition (C, R, T, CR, CT, RT, COH, COHT, and B) on all time terms. The model also included the random effects of by-participant and by-item intercept. The significance of the main effects and interactions of the models were obtained *via* likelihood ratio comparisons with the change in log-likelihood distributed as χ^2^. Results of the competitor model and the target model are presented in Tables 1, 2, respectively. *Post hoc* comparisons for these two models were performed using the *glht* function with Bonferroni adjustment in the Multcomp package in R (Hothorn et al., 2008).

**TABLE 1:** Fixed effects for the model of looks to competitors.

Fixed effects	χ^2^	D*f*	*p*
Linear	7,563.82	1	<0.001
Quadratic	434.86	1	<0.001
Cubic	2,989.82	1	<0.001
Quartic	842.76	1	<0.001
Condition: intercept	16.78	8	<0.05
Condition: linear	218.90	8	<0.001
Condition: quadratic	230.61	8	<0.001
Condition: cubic	233.8	8	<0.001
Condition: quartic	26.02	8	<0.01

**TABLE 2:** Fixed effects for the model of looks to targets.

Fixed effects	χ^2^	D*f*	*p*
Linear	16,938.00	1	<0.001
Quadratic	11,933.15	1	<0.001
Cubic	2,412.98	1	<0.001
Quartic	953.39	1	<0.001
Condition: intercept	6.05	8	n.s.
Condition: linear	77.35	8	<0.001
Condition: quadratic	108.38	8	<0.001
Condition: cubic	33.73	8	<0.001
Condition: quartic	20.60	8	<0.01

In addition, for each competitor condition, fixation proportions to competitors and distractors were analyzed using a four-order orthogonal polynomial with fixed effects of Role (competitor and distractor) on all time terms. Each model also included random effects of participants on all time terms and by-item random intercept. The *P*-values in all models were estimated using the normal approximation for the *t*-values.

## Results

### Behavior Results

The mean reaction time (measured from the onset of auditory stimuli to participants’ response) and response accuracy for the nine conditions are shown in Table 3. The accuracy is very high across conditions and only one error was made in the RT condition. The reaction time did not show a significant difference across conditions, since the main effect of Condition was not significant in a repeated-measures ANOVA [*F* (8,19) = 0.77, *p* = 0.63].

**TABLE 3:** Mean reaction time and mean percent accuracy for the click response.

Condition	Reaction time (ms)	Percent accuracy
B	1,275.1 (28.0)	100 (0)
C	1,282.1 (35.4)	100 (0)
R	1,278.0 (16.5)	100 (0)
T	1,294.1 (17.5)	100 (0)
CR	1,281.3 (14.2)	100 (0)
CT	1,252.1 (14.1)	100 (0)
RT	1,255.8 (13.7)	99.8 (0.002)
COH	1,277.0 (14.8)	100 (0)
COHT	1,273.5 (15.2)	100 (0)

*Values in parentheses represent standard errors.*

### Eye-Movement Data

For all eight phonological overlapping conditions, the fixation dwelling time in four interest areas was not significantly different during the preview section (all *p* values > 0.05), suggesting that participants approximately equally distributed their attention to the target, competitor, and two distractors, and there was no systematic bias caused by pictorial information before the auditory stimulus was presented.

For the model of fixations to competitors across all conditions, there was a main effect of Condition on all time terms (Table 1). *Post hoc* multi-comparison results were reported in this section when comparisons were made across different conditions. Similarly, for the model of fixations to targets across all conditions, the effect of Condition on the intercept was not significant, but the effect of Condition on the linear, quadratic, cubic, and quartic terms were all statistically significant (Table 2). *Post hoc* multi-comparison results were reported later in this section when fixations to targets of each condition were compared to the baseline condition.

To examine the specific role of the consonant, rime, and tone, we first compared the competitor looks in each condition versus the fixations to the phonologically unrelated distractors, and then the looks to target in each condition were compared with the baseline. The roles of the combinations of the components were investigated with the same procedure.

**FIGURE 3:**
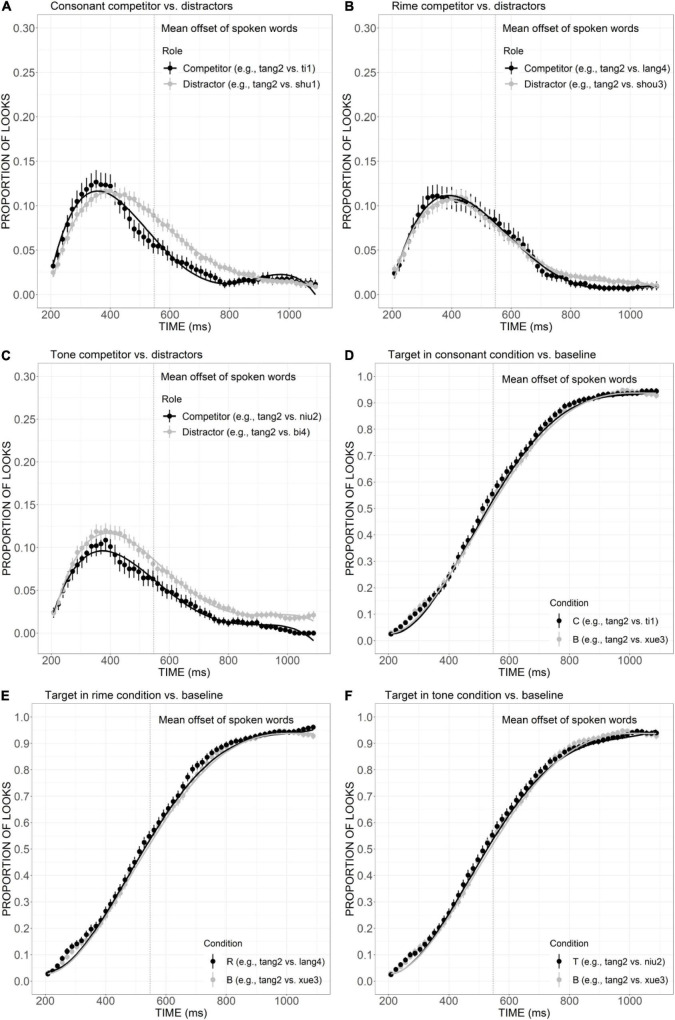
Competitor **(A–C)** and target **(D–F)** fixations in the C, R, and T conditions. The competitor curves are plotted against the phonologically unrelated distractors and the target curves are plotted against the baseline competitors (B) which have no phonological overlap with the targets. The points refer to mean proportions of looks to targets and competitors across participants and items. The lines represent model fits of growth curve analysis.

**FIGURE 4:**
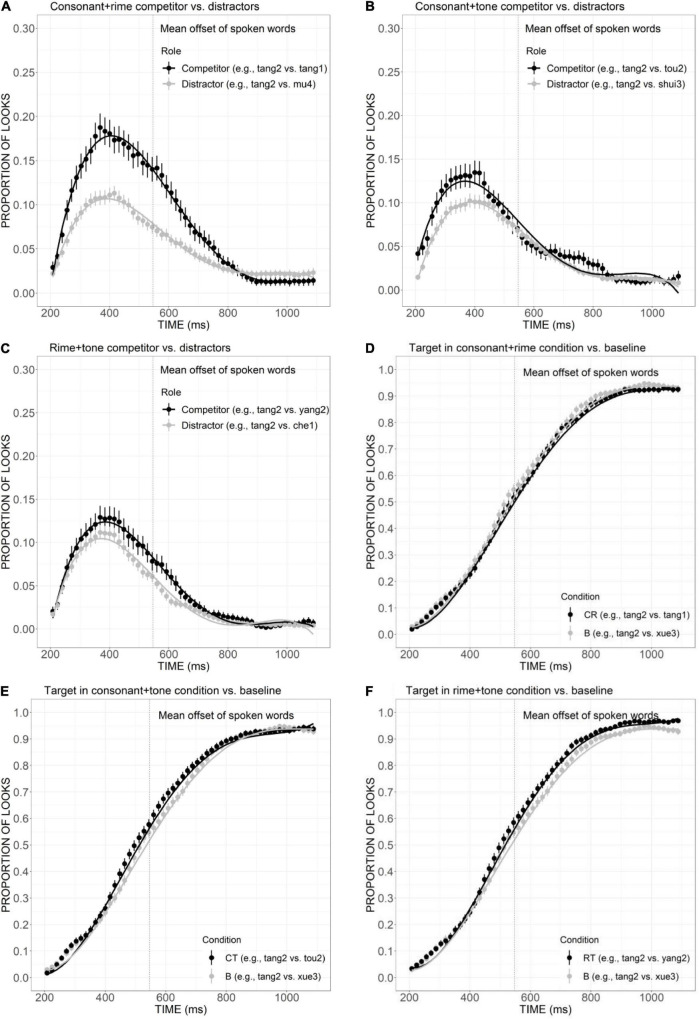
Competitor **(A–C)** and target **(D–F)** fixations in the CR, CT, and RT conditions. The competitor curves are plotted against the phonologically unrelated distractors and the target curves are plotted against the baseline competitors (B) which have no phonological overlap with the targets. The points refer to mean proportions of looks to targets and competitors across participants and items. The lines represent model fits of growth curve analysis.

The mean proportion of looks to the competitor was plotted against the distractors in the consonant, rime, and tone conditions (Figure 3). As shown in Figure 3A, the curve of consonant competitor exhibited an earlier and higher peak at approximately 350 ms, compared with the distractor curve which reached its maximum after 400 ms. That is, the competitor curve rose and declined much more steeply than the distractor, as confirmed by significant differences in quadratic (Est. = –0.09, *t* = –8.75, *p* < 0.001), cubic (Est. = 0.02, *t* = 2.23, *p* < 0.001), and quartic (Est. = 0.05, *t* = 4.58, *p* < 0.001) components, indicating that the overlap in consonant made the competitor activated earlier and to a greater extent than the distractors. As depicted in Figure 4B, the rime competitor also demonstrated a slightly earlier peak compared with distractors, indexed by a significant difference in linear component (Est. = 0.03, *t* = 2.54, *p* < 0.05). During 500–700 ms, there was a tendency of more looks to the competitor than distractors, which is indicative of continuous activation of the competitor with rime overlap, compared with the sharp decline observed in the consonant competitor. The fixations to tone competitor (Figure 3C), however, were significantly less than the distractors during the whole processing time, as confirmed by significant difference in the intercept (Est. = 0.02, *t* = 3.14, *p* < 0.01), quadratic (Est. = –0.02, *t* = –2.16, *p* < 0.05), and cubic (Est. = 0.04, *t* = 3.86, *p* < 0.001) terms, suggesting that overlap only in tone was not sufficient to distract the attention of the participants.

As shown in Figures 3D–F, in all conditions, the proportion of looks to target as a function of time gradually ramped up and reached the maximum of more than 90% around 1,000 ms, exhibiting a sigmoidal curve. The curves of target look exhibit great overlapping with baseline in the consonant, rime, and tone conditions, with no significant effect found in the *post hoc* comparison, indicating the presence of these competitors matching the target with only one aspect cannot cause a significant delay in the activation of the target.

The mean proportion of looks to competitor was plotted against the distractors in CT, RT, and CR conditions in Figures 4A–C. The strongest activation had been observed for the CR competitor (Figure 4A). It attracted much greater looks than distractors from 200 ms to approximately 850 ms, as confirmed by the significant differences in the intercept (Est. = –0.03, *t* = –3.93, *p* < 0.001), linear (Est. = 0.18, *t* = 14.02, *p* < 0.001), quadratic (Est. = 0.09, *t* = 7.12, *p* < 0.001), and cubic (Est. = –0.12, *t* = 9.13, *p* < 0.001) components. CT competitors (Figure 4B) also received higher proportion of looks compared to distractors from 200 ms to the offset of the auditory stimulus around 560 ms, as confirmed by the significant effect in linear (Est. = 0.07, *t* = 6.16, *p* < 0.001), quadratic (Est. = –0.03, *t* = –2.34, *p* < 0.05), and quartic (Est. = 0.02, *t* = 2.06, *p* < 0.05) components. Similarly, more fixations were also launched to RT competitors than distractors (Figure 4C), as indexed by significant differences in linear (Est. = 0.04, *t* = 3.42, *p* < 0.001), quadratic (Est. = 0.03, *t* = 2.81, *p* < 0.01), and cubic (Est. = 0.04, *t* =–4.18, *p* < 0.001) components. Compared with CT competitors, the activation of RT competitor lasted for a longer duration, which can be observed across the whole processing time, from 200 ms to approximately 900 ms.

The curves of the target looks in the CR, CT, and RT conditions were plotted against baseline (Figures 4D–F). The target in the CR condition (Figure 4D) received more fixations from 400 ms to the end of the trial, but the difference failed to reach statistical significance. The curve of target looks in the CT condition (Figure 4E) cannot be distinguished from baseline curve from 200 to 400 ms, but attracted more fixations from 400 ms to around 800 ms, which was reflected in significant differences in the quadratic term (Est. = –0.11, *z* =–5.29, *p* < 0.001). In the RT condition (Figure 4F), the target also received more fixations than baseline after the end of the sound stimulus, which is indicated by the significant difference in the quadratic term (Est. = –0.08, *z* = –3.84, *p* < 0.05). Such differences between the CT and RT targets versus baseline were opposite to the expected pattern and might indicate that the presence of CT and RT competitors did not exert strong interferences to the targets.

To sum up, among the competitor overlapped with the target in one sub-syllabic item, consonant competitors were activated adequately to compete with the targets compared with the phonologically unrelated distractors. Rime competitors were also activated to a smaller extent. Tone competitors, however, could not be activated effectively as a candidate for lexical competition. Furthermore, all the three competitors sharing two sub-syllabic items with the target (CR, CT, and RT competitors) attracted more fixations compared to distractors, suggesting an interference role played by them to compete for recognition.

**FIGURE 5:**
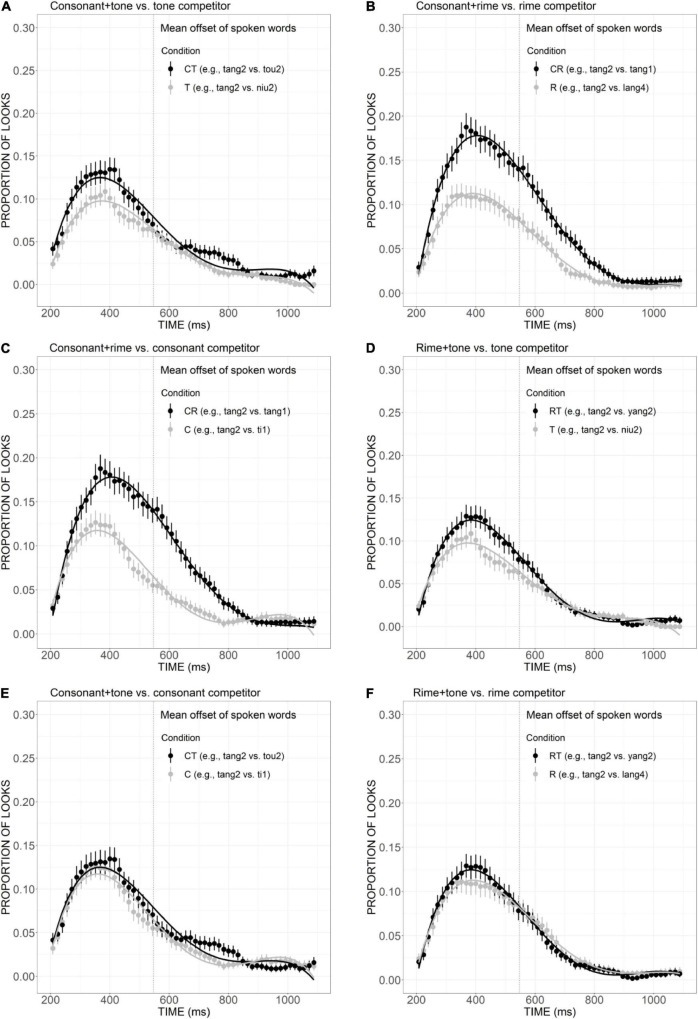
Competitor fixations in the CT versus T conditions **(A)**, CR versus R conditions **(B)**, CR versus C conditions **(C)**, RT versus T conditions **(D)**, CT versus C conditions **(E)**, and RT versus R conditions **(F)**. The points refer to mean proportions of looks to competitors across participants and items. The lines represent model fits of growth curve analysis.

In order to illustrate the relative contribution of consonant, rime, and tone in lexical access, we further compared the competitor effects across conditions. First, to examine the role of the consonant in spoken word recognition, comparisons were made between the T condition versus the CT condition, as well as the R condition versus the CR condition. As revealed in Figure 5A, there was a clear tendency of greater looks to CT competitors than T competitors from the beginning to nearly the offset of the auditory stimuli, although the statistic results only suggested a marginal difference in linear component (Est. = 0.05, *z* = 3.55, *p* = 0.07). The significantly more fixations to the CR competitor than the R competitor (Figure 5B), as indexed by significant differences in the linear (Est. = 0.13, *z* = 9.34, *p* < 0.001), quadratic (Est. = 0.09, *z* = 6.32, *p* < 0.001), and cubic (Est. = –0.09, *z* =–6.70, *p* < 0.001) components, suggested the additional overlap in consonant did strengthen the activation effect to a greater extent. Second, the role of rime can be shown through the comparisons of C versus CR conditions and T versus RT conditions. Figure 5C presents a clear pattern of greater looks to CR competitor than C, supported by significant results in linear (Est. = –0.12, *z* =–9.17, *p* < 0.001), quadratic (Est. = –0.16, *z* =–11.56, *p* < 0.001), and cubic (Est. = 0.13, *z* = 9.36, *p* < 0.001) components. Statistically significant difference was also found between T and RT conditions in cubic component (Est. = –0.07, *z* =–5.45, *p* < 0.001; Figure 5D). Such differences are indicative of the important role of rime in facilitating word activation when it is combined with tone and consonant. Finally, to investigate the contribution of the tone, of interest were the comparisons of C versus CT, and R versus RT conditions. As shown in Figure 5E, the curves of C and CT competitors presented a great amount of overlap, especially for the initial part of the processing, from the beginning to the end of the auditory stimulus. The statistical analysis also supported the non-significant difference between the two conditions. Similarly, although the curve of RT competitor showed a tendency of higher than R competitor around 400 ms (Figure 5F), the difference failed to reach statistical significance. Collectively, the additional overlap in consonant and rime made the originally less activated words more competitive, which suggested their important role in strengthening the interference effect. Competitors with additional tone overlap failed to draw more fixations, which may indicate a less important role of tone in lexical activation compared with consonant and rime.

**FIGURE 6:**
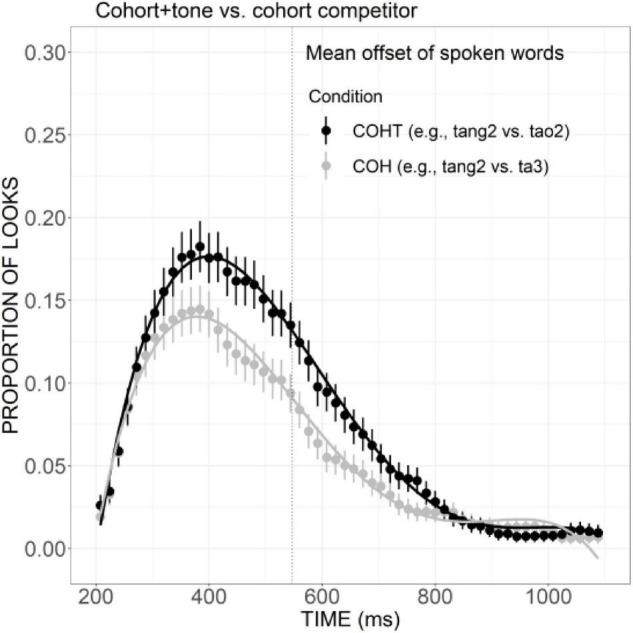
Competitor fixations in the COH versus COHT conditions. The points refer to mean proportions of looks to competitors across participants and items. The lines represent model fits of growth curve analysis.

But it should be noted that, with the increase in phonological similarity, the additional overlap in tone can effectively facilitate the activation of cohort plus tone competitor (e.g., *ta3* “tower”) compared with cohort competitor (e.g., *tao2* “peach”) (Figure 6), as indexed by significant differences in the linear (Est. =–0.07, *z* =–4.95, *p* < 0.001), quadratic (Est. =–0.07, *z* =–5.01, *p* < 0.001), and cubic (Est. = 0.08, *z* = 6.06, *p* < 0.001) terms.

**FIGURE 7:**
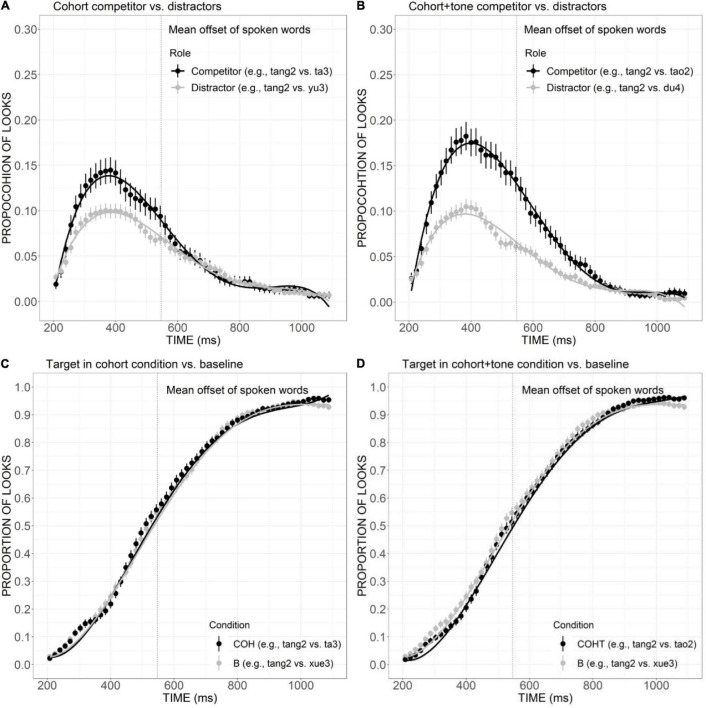
Competitor **(A,B)** and target **(C,D)** fixations in the COH and COHT conditions. The competitor curves are plotted against the phonologically unrelated distractors and the target curves are plotted against the baseline competitors (B) which have no phonological overlap with the targets. The points refer to mean proportions of looks to targets and competitors across participants and items. The lines represent model fits of growth curve analysis.

To investigate the relative weighting of cohorts versus rimes, we first examined the proportion of looks to COH and COHT competitors relative to phonologically unrelated distractors and then compared the target looks in these two conditions with the baseline. After that, the competitive effects of COH versus R conditions, as well as COHT versus RT conditions were compared. As shown in Figure 7A, compared with the distractors, much more fixations were launched to cohort competitors during the time window from 200 to 600 ms, as suggested by significant differences in intercept (Est. =–0.01, *t* =–2.50, *p* < 0.05), linear (Est. = 0.08, *t* = 7.40, *p* < 0.001), cubic (Est. =–0.06, *t* =–5.33, *p* < 0.001), and quartic (Est. = 0.06, *t* = 4.97, *p* < 0.001) terms. A similar effect was observed in the COHT condition (Figure 7B), which was stronger and more prolonged, with the competitor curve higher than distractors from the beginning of the trial to around 800 ms. This was confirmed by significant results in all components: intercept (Est. =–0.03, *t* =–5.62, *p* < 0.001), linear (Est. = 0.15, *t* = 12.48, *p* < 0.001), quadratic (Est. = 0.08, *t* = 6.32, *p* < 0.001), cubic (Est. =–0.14, *t* =–11.65, *p* < 0.001), and quartic (Est. = 0.03, *t* = 2.54, *p* < 0.05). With respect to target looks, the curve of target look in the COH condition (Figure 7C) presented a shallower slope compared with the baseline in the initial proportion of the trial, but such difference was not statistically significant. The target in the COHT condition (Figure 7D) also received a lower proportion of looks than the baseline. The effect prevailed from 200 to around 500 ms and lasted to almost the end of the trial (linear component: Est. = 0.10, *z* = 4.92, *p* < 0.001), suggesting that the presence of COHT competitors did interfere with the activation of targets.

**FIGURE 8:**
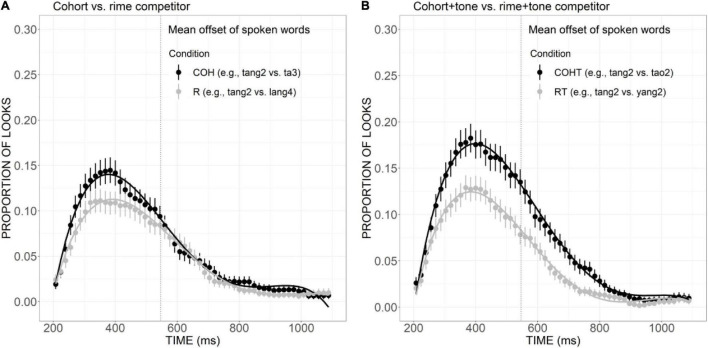
Competitor fixations in the COH versus R conditions **(A)** and COHT versus RT conditions **(B)**. The points refer to mean proportions of looks to competitors across participants and items. The lines represent model fits of growth curve analysis.

As presented in Figure 8A, the curve of COH competitor generally showed a steeper rising tendency in the initial proportion of the trial and declined rather rapidly after the peak at a little earlier than 400 ms. The proportion of looks for R competitor, on the other hand, rose slowly and reached a plateau from ca. 320 to 500 ms, and then it decreased in a slower way, suggesting a weaker but more prolonged competitive effect compared with the COH condition. A significant difference in the linear component was found between these two conditions (Est. = 0.05, *z* = 3.75, *p* < 0.05). Compared with the COHT competitor, the RT competitor also showed a weaker competitive effect (Figure 8B), as indexed by significant differences in the linear (Est. = 0.09, *z* = 6.90, *p* < 0.001), quadratic (Est. = 0.07, *z* = 5.42, *p* < 0.001), and cubic (Est. =–0.07, *z* =–5.41, *p* < 0.001) components.

## Discussion

### The Specific Role of Consonant, Rime, and Tone in Mandarin Monosyllabic Word Recognition

The relative contribution of sub-syllabic components was demonstrated by a close examination of the consonant, rime, and tone conditions. As is evident in Figure 3, in comparison with the distractors, the consonant overlap (e.g., *ti1* “ladder”) attracted more fixations and showed a competitive effect in an early stage of processing. The rime competitor (e.g., *lang4* “wave”) also received a slightly higher proportion of looks compared with the distractors, which is indicative of its role as a competitive candidate. Differently, the competitor overlapping the target only in tone (e.g., *niu2* “cow”) was not adequately activated to compete for lexical access. This indicates tonal information’s less important role compared with consonant and rime. Moreover, the competitive effect generally becomes stronger as the degree of phonological overlap with the target word increases: competitors overlapping the target in two sub-syllabic components were generally more activated than competitors only sharing one component. More specifically, the relative weighting of these sub-syllabic items in combination was obtained by comparisons across different competitor conditions. As is evident in Figure 5, with the additional overlap in the consonant, consonant plus tone competitor exhibited a stronger activation effect than tone competitor. Consonant plus rime competitor was also activated to a larger extent compared with rime competitor, which is indicative of a great contribution of the consonant in strengthening the activation of lexical candidates. A similar contribution has also been observed for rime, as suggested by a stronger activation of consonant plus rime competitor than the consonant competitor, as well as a stronger activation of rime plus tone competitor than tone competitor. The additional overlap in tone, however, failed to exert a significantly more activation effect, and this may indicate that just like tone competitors cannot attract more fixations than unrelated distractors, the contribution of tone is also not obvious when it appears in the combination with consonant or rime. This finding is consistent with prior results from Tong et al. (2014) and Wiener and Turnbull (2016) that Mandarin speakers rely more on vowels and consonants in lexical access.

However, with the increase in phonological similarity, the additional overlap in tone can effectively facilitate the activation of the cohort plus tone competitor (e.g., *ta3* “tower”) compared with the cohort competitor (e.g., *tao2* “peach”) (Figure 6). This may suggest that when the overlap with the expectation accumulated to a certain degree, any information added may trigger great activation. The cohort competitors already matched the targets in the consonant and the initial proportion of rime, the additional match in tone information becomes crucial in making the competitor a highly potential candidate.

### The Incremental Processing of Mandarin Monosyllabic Words

Whether monosyllabic words in Mandarin are processed incrementally or more holistically was addressed by examining the magnitude and time course of activation in each competitor condition. If the syllable is processed in a holistic manner, then only neighbors differing from the target in one phoneme (i.e., the CR, RT, and COHT competitors) will be activated adequately, while the immediate activation of partially overlapping competitors (e.g., the consonant competitor and the rime competitor) will indicate the processing is incremental.

**FIGURE 9:**
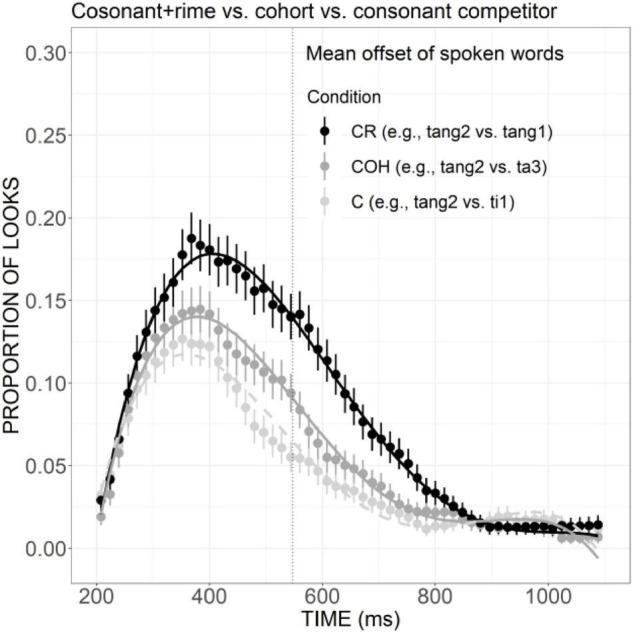
Competitor fixations in the C, COH, and CR conditions. The points refer to mean proportions of looks to competitors across participants and items. The lines represent model fits of growth curve analysis.

According to the results, competitors overlapping the target only in consonant (e.g., *ti1*, ladder) and only in rime (e.g., *lang4*, wave) can be activated effectively, as measured by an earlier and higher peak of fixation curve for the consonant competitor and slightly more fixations than the unrelated distractors for rime competitors. With the increase of goodness of fit between the expected target and competitor, the activation of the competitors sharing two sub-syllabic components are significantly more activated than distractors and competitors matching the target in only one component. For example, competitors overlapping the target in consonant and tone were more activated compared to tone competitors. More activation was also found for the consonant plus rime competitor than the competitor matching the target only in rime (Figure 5). This gradation of activation as a function of the phonological similarity between target and competitors is illustrated more clearly in Figure 9. Compared with the consonant competitor, the match of an extra phoneme in the cohort condition led to a shallower declining slope and significantly more fixations, and a similar pattern can also be observed between consonant and rime competitor versus cohort competitor, indicating that lexical access takes place immediately with receipt of a minimal amount of acoustic information and the activation is updated incrementally with the competitors bearing more phonological similarity be activated to a larger extent. These results are in line with other recent findings (e.g., Ho et al., 2019) and the prediction of continuous mapping models (such as the TRACE model), which assume that as speech input unfolds over time, it is continuously mapped onto potential lexical representations which compete for recognition.

### The Weighting of Cohorts and Rimes in Mandarin Monosyllabic Word Processing

In the present study, it was found that both cohort competitors matching targets in initial consonant and the initial phonemes of rime, and cohort plus tone competitor were activated to compete for lexical recognition, as measured by significantly more fixations than unrelated distractors. Likewise, the rime and rime plus tone competitors were also more activated than unrelated distractors, although the effect for rime competitors was weaker. The differences between cohort and rime competition have also been revealed. Cohort activation, as suggested by the proportion of looks to cohort competitor, exhibited a steeper rising slope and had a higher peak than rime activation. A similar difference has also been found between the cohort plus tone and the rime plus tone condition with a larger effect. Taken together, in Mandarin Chinese, both cohort and rime overlaps can exert an activation effect, but the effect size of rime is weaker compared with the cohort. Moreover, cohort versus rime competitors is not temporally distinct from one another.

In English, eye-tracking studies have confirmed that for a target word such as *beaker*, both cohort competitor *beetle* and rhyme competitor *speaker* can be activated to compete for recognition, but the rhyme competitor was activated later and to a smaller extent compared to the cohort competitor. The results of the current research are partially in line with the patterns in English since a smaller size of competitive effect has been found for rime than cohort competitors. But the temporal delay in the activation of rime competitors was not obvious in Mandarin, which may suggest that rimes play a more important role than they do in English: a role that is equivalent to the role of cohorts at least in the temporal domain.

These results also contribute to clarifying the mixed findings in previous studies on the weighing of cohorts versus rimes in Mandarin. The competitive effect for rimes found in the present study is compatible with the findings in Zhao et al. (2011) which inferred that both cohorts and rimes are important in word recognition and are not temporally distinct. The current results, however, are inconsistent with the findings in Malins and Joanisse (2010) which failed to show competitive effects for rhyming words and thus indicative of the weak influence of rime competitors on Mandarin word recognition compared to that in English. These results also provide strong support for the assumptions of continuous mapping models such as TRACE, that is, lexical candidates bearing sufficient similarity to the target can become activated to compete for recognition, whether or not they share the same onset.

### Implications for Models of Spoken Word Recognition

**FIGURE 10:**
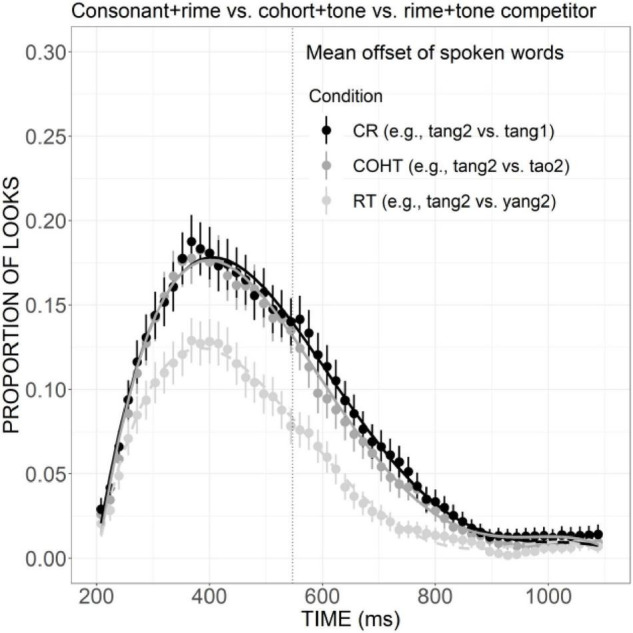
Competitor fixations in the CR, COHT, and RT conditions. The points refer to mean proportions of looks to competitors across participants and items. The lines represent model fits of growth curve analysis.

The current findings have several implications for modeling monosyllabic word processing in Mandarin Chinese. First, the present results seem to be inconsistent with NAM, which emphasizes the importance of global similarity among candidates and targets and treats words that differ with the input by one phoneme equally regardless of the position of the mismatch (Luce and Pisoni, 1998). According to this model, all the competitors that differed from the target in only one phoneme (i.e., consonant plus rime competitors, cohort plus tone competitors, and rime plus tone competitors) should be activated to a similar degree. Our results showed that for a target word (e.g., *tang2* candy) both consonant plus rime competitors (e.g., *tang1* soup) and cohort plus tone competitors (e.g., *tao2* peach) exhibited a comparable great effect of activation, but the rime plus tone competitors (e.g., *yang2* sheep) were activated to a much less degree compared with the other “neighbors,” which is at odds with the prediction of NAM (Figure 10). The immediate use of consonant and rime information to modulate the dynamics of lexical competition found in the current study clearly suggests an incremental nature, which is also different from the global processing view of NAM and the holistic fashion of Mandarin word processing proposed in previous studies (e.g., Zhao et al., 2011).

Instead, the current data are more compatible with the online processing models (e.g., the TRACE model and the Cohort model). The Cohort model emphasizes the importance of onset match during the mapping between the input and the representations, highlighting the temporal sequence of word recognition. Since both words sharing word-initial information (i.e., cohort and cohort plus tone competitors) and words sharing word-final information (i.e., rime and rime plus tone competitors) exert adequate competitive effects in our data, it seems that the TRACE model may be the optimal one among existing models to account for the current data.

Second, in terms of incorporating tones into the existing models, in line with previous modified models (e.g., Malins and Joanisse, 2010; Shuai and Malins, 2017), we assume that the tonal features need to be specified at the initial feature level and the “toneme” node should be added beside the phoneme level. Importantly, current results suggest a weaker role for tone match in facilitating the competitive effect of phonologically similar competitors. Different from consonant and rime, competitors with only tone overlap (e.g., target: *tang2* “candy,” tone competitor: *niu2* “cow”) failed to show competitive effects compared with phonologically unrelated distractors. For competitors matching the target in one sub-syllabic component (e.g., consonant competitor: *ti1* “ladder”), the additional match in tone information (e.g., consonant plus tone competitor: *tou2* “head”) also cannot effectively strengthen the competitive effect, leading to the conclusion of the relatively less important role of tones. It is noteworthy that, competitors that overlap the target in the consonant, initial part of rime, and tone (cohort plus tone competitor: e.g., *tao2* “peach”) can be activated to a larger extent compared with competitors that overlap the target only in consonant and the onset part of rime (cohort competitor: e.g., *ta3* “tower”), which suggests that the overlap in tone does contribute in amplifying the competitive effect when the similarity between the competitor and target has reached a certain degree.

Lastly, in line with the TRACE model, the current data suggest a gradation of activation based on the acoustic similarity between targets and candidates. What’s more, such gradation may reflect the strategy of native speakers shaped by the characteristics of information values of different combinations of consonant, rime, and tone. The current data reveal that competitors overlapping the target only in one sub-syllabic item, namely consonant, rime, and tone competitors, display the least activation, and the competitors with two or more aspects overlap can be activated to a stronger degree, with consonant plus rime and cohort plus tone competitors most strongly activated. These results are generally consistent with the TTRACE model proposed by Tong et al. (2014), which could depict the different levels of activation based on the inter-word interaction of different sub-syllabic items. Our data also generally conform to the characteristics of information values of Mandarin interior syllabic structures. According to the information-theoretic methods (Garner and Miller, 1988; Tong et al., 2008), in the context of word recognition, the ability of a given signal to constrain recognition is related to its probability of occurring in a corresponding communication system. Signals with a lower probability of occurrence have a greater ability to exclude alternative candidates and thus enjoy larger information value, that is, the informational value of a signal in a communication system is inversely proportional to its probability of occurrence.

Following the method used in Tong et al. (2008), the equivalence class size (ECS) of different types of competitors in the current study has been calculated using the database by Tsai (2000), which includes 13,060 Mandarin characters and 1,251 unique segment-tone combinations.

The ECS is used to quantify the amount of information carried by each sub-syllabic structure. For example, the ECS of the consonant /t/ is 52, which means that within the 1,251 unique segment-tone combinations, there are 52 combinations that begin with this consonant. Knowing the initial consonant of a segment-tone combination is /t/ can effectively increase the probability of correctly identifying that combination from 1/1,251 to 1/52. Similarly, knowing the consonant and rime of a segment-tone combination is /ti/ (with ECS of 4) can greatly increase the probability of correct identification from chance probability (i.e., 1/1,251) to 1/4. That is to say, informational value is inversely related to ECS: the smaller the ECS value, the more information is carried by the combination. The average ECS for each type of competitor is presented in Table 4.

**TABLE 4:** Mean equivalence class size values and standard deviations for different competitor conditions.

Condition	Mean	SD
C	47.65	12.63
R	32.79	18.56
T	310.50	38.06
CR	3.09	0.94
CT	12.04	4.30
RT	8.25	4.80
COH	9.11	6.63
COHT	2.60	1.75

One-way ANOVA suggested that competitors differed significantly in their information value [*F* (7,1552) = 2,769, *p* < 0.001]. *Post hoc* pairwise comparisons with Bonferroni adjustment further showed that both consonant competitors and rime competitors had significantly smaller ECS values than that of tone competitors (all *p-*values < 0.001), indicating an overlap in consonant or rime are more informative than overlap in tone. Moreover, the ECS values of the consonant plus rime competitor and the cohort plus tone competitor were significantly smaller than that of all the other competitors (all *p-*values < 0.001), which suggests these two kinds of competitors are the most informative ones. These results of statistical analyses are quite in line with those of the current eye-tracking data, reflecting native speakers may have formed a strategy of being more attentive to candidates that are more informative.

## Conclusion

The present study examined the time course of Mandarin monosyllabic word recognition using an eye-tracking experiment with a visual world paradigm. The issues of interest are the relative contribution of sub-syllabic components and their combinations in word processing, whether Mandarin syllable is processed incrementally or in a more holistic manner, and the weighting of onset and rime in Mandarin word recognition. Results revealed that both consonant and rime overlap can generate adequate competitive effects. The contribution of tone seemed to be weaker compared to segments, but it can strengthen the competitive effect when it was added to a candidate which already bore much acoustic similarity with the expected target. Furthermore, the gradation of activation based on the acoustic similarity between targets and competitors suggested an incremental way of processing, which is in line with the continuous mapping models. Also, both cohorts and rimes were activated to compete for lexical access, which is also consistent with the assumption of continuous mapping models, that lexical candidates with sufficient similarity to the target can be activated for recognition, whether sharing the same onset or not. The activation patterns of different partially similar competitors were also in line with the informative characteristics of different sub-syllabic structures in Mandarin, which may reflect a strategy of native speakers shaped by their language experiences.

## Data Availability Statement

The raw data supporting the conclusions of this article will be made available by the authors, without undue reservation.

## Ethics Statement

Ethical review and approval was not required for the study on human participants in accordance with the local legislation and institutional requirements. The patients/participants provided their written informed consent to participate in this study.

## Author Contributions

TZ: experimental design and wrote the manuscript. YL: data collection and statistical analysis. HZ: data collection. All authors contributed to the article and approved the submitted version.

## Conflict of Interest

The authors declare that the research was conducted in the absence of any commercial or financial relationships that could be construed as a potential conflict of interest.

## Publisher’s Note

All claims expressed in this article are solely those of the authors and do not necessarily represent those of their affiliated organizations, or those of the publisher, the editors and the reviewers. Any product that may be evaluated in this article, or claim that may be made by its manufacturer, is not guaranteed or endorsed by the publisher.

## References

[B1] Allopenna P. D. Magnuson J. S. Tanenhaus M. K. (1998). Tracking the time course of spoken word recognition using eye movements: evidence for continuous mapping models. *J. Mem. Lang.* 38 419–439.

[B2] Bates D. Maechler M. Bolker B. Walker S. (2014). *lme4**: Linear Mixed-Effects Models Using Eigen and S4. R package version 1.1-7.*

[B3] Bent T. (2005). *Perception and Production of Non-Native Prosodic Categories.* Doctoral dissertation. Evanston, IL: Northwestern University.

[B4] Chen J.-Y. Chen T.-M. Dell G. S. (2002). Word-form encoding in Mandarin Chinese as assessed by the implicit priming task. *J. Mem. Lang.* 46 751–781. 10.1016/j.actpsy.2012.11.011 23261962

[B5] Chen Y. Gussenhoven C. (2008). Emphasis and tonal implementation in Standard Chinese. *J. Phonetics.* 36 724–746.

[B6] Chen T.-M. Chen J.-Y. (2013). The syllable as the proximate unit in Mandarin Chinese word production: an intrinsic or accidental property of the production system? *Psychon. Bull. Rev.* 20 154–162. 10.3758/s13423-012-0326-7 23065764

[B7] Chen W. Chao P. Chang Y. Hsu C. Lee C. (2016). Effects of orthographic consistency and homophone density on Chinese spoken word recognition. *Brain Lang.* 157 51–62. 10.1016/j.bandl.2016.04.005 27174851

[B8] Choi W. Tong X. Gu F. Tong X. Wong L. (2017). On the early neural perceptual integrality of tones and vowels. *J. Neurolinguistics* 41 11–23. 10.1016/j.jneuroling.2016.09.003

[B9] Connell K. Tremblay A. Zhang J. (2016). “The timing of acoustic vs. perceptual availability of segmental and suprasegmental information,” in *Proceedings of the 5th International Symposium on Tonal Aspects of Languages*, New York, NY, 99–102.

[B10] Cutler A. Chen H. (1997). Lexical tone in cantonese spoken-word processing. *Percept. Psychophys.* 59 165–179. 10.3758/bf03211886 9055613

[B11] Cutler A. Dahan D. Van Donselaar W. (1997). Prosody in the comprehension of spoken language: a literature review. *Lang. Speech* 40(Pt 2), 141–201. 10.1177/002383099704000203 9509577

[B12] Dahan D. Gaskell M. G. (2007). The temporal dynamics of ambiguity resolution: evidence from spoken-word recognition. *J. Mem. Lang.* 34 269–284. 10.1016/j.jml.2007.01.001 18071581 PMC2128696

[B13] Davis C. Schoknecht C. Kim J. Burnham D. (2016). The time course for processing vowels and lexical tones: reading aloud Thai words. *Lang. Speech* 59 196–218. 10.1177/0023830915586033 27363253

[B14] Desroches A. S. Newman R. L. Joanissse M. F. (2009). Investigating the time course of spoken word recognition: electrophysiological evidence for the influence of phonological similarity. *J. Cogn. Neurosci.* 21 1893–1906. 18855555 10.1162/jocn.2008.21142 PMC3965566

[B15] Duanmu S. (2000). *The Phonology of Standard Chinese.* Oxford: Oxford University Press.

[B16] Duanmu S. (2007). *The Phonology of Standard Chinese*, 2nd Edn. New York: Oxford University Press.

[B17] Frauenfelder U. H. Tyler L. K. (1987). The process of spoken word recognition: an introduction. *Cognition* 25 1–20. 10.1016/0010-0277(87)90002-3 3581722

[B18] Gao X. Yan T. T. Tang D. L. Huang T. Shu H. Nan Y. (2019). What makes lexical tone special: a reverse accessing model for tonal speech perception. *Front. Psychol.* 10:2830. 10.3389/fpsyg.2019.02830 31920863 PMC6930229

[B19] Garner W. R. Miller A. (1988). “The contribution of information theory to psychology,” in *The Making of Cognitive Science: Essays in Honour of George*, ed. Hirst W. (Cambridge, UK: Cambridge University Press).

[B20] Hallet P. E. (1986). “Eye movements,” in *Handbook of Perception and Human Performance*, eds Boff K. Kaufman L. Thomas J. (New York: Wiley).

[B21] Ho A. Boshra R. Schmidtke D. Oralova G. Moro A. L. Service E. (2019). Electrophysiological evidence for the integral nature of tone in mandarin spoken word recognition. *Neuropsychologia* 131 325–332. 10.1016/j.neuropsychologia.2019.05.031 31185227

[B22] Hothorn T. Bretz F. Westfall P. (2008). Simultaneous inference in general parametric models. *Biometrical J.* 50 346–363. 10.1002/bimj.200810425 18481363

[B23] Howie J. M. (1974). On the domain of tone in Mandarin. *Phonetica* 30 129–148. 10.1159/000259484

[B24] Hu J. Gao S. Ma W. Yao D. (2012). Dissociation of tone and vowel processing in Mandarin idioms. *Psychophysiology* 49 1179–1190. 10.1111/j.1469-8986.2012.01406.x 22748083

[B25] Huettig F. Rommers J. Meyer A. S. (2011). Using the visual world paradigm to study language processing: a review and critical evaluation. *Acta Psychol.* 137 151–171. 10.1016/j.actpsy.2010.11.003 21288498

[B26] Jesse A. Poellmann K. Kong Y. Y. (2017). English listeners use suprasegmental cues to lexical stress early during spoken-word recognition. *J. Speech Lang. Hear Res.* 60 190–198. 10.1044/2016_JSLHR-H-15-0340 28056135 PMC5533556

[B27] Kamide Y. Altmann G. Henderson J. M. Ferreira F. (2004). “Now you see it, now you don’t: mediating the mapping between language and the visual world,” in *The Interface of Language Vision & Action Eye Movements & the Visual World*, eds Henderson J. M. Ferreira F. (Hove: Psychology Press).

[B28] Li Q. (2016). *The Production and Perception of Tonal Variation: Evidence from Tianjin Mandarin.* Utrecht: LOT.

[B29] Ling W. Grüter T. (2020). From sounds to words: the relation between phonological and lexical processing of tone in L2 Mandarin. *Sec. Lang. Res.* 026765832094154. 10.1177/0267658320941546

[B30] Luce P. A. Pisoni D. B. (1998). Recognizing spoken words: the neighborhood activation model. *Ear Hear.* 19 1–36. 10.1097/00003446-199802000-00001 9504270 PMC3467695

[B31] Ma W. Zhou P. Singh L. Gao L. (2017). Spoken word recognition in young tone language learners: age-dependent effects of segmental and suprasegmental variation. *Cognition* 159 139–155. 10.1016/j.cognition.2016.11.011 27951429

[B32] Malins J. G. Joanisse M. F. (2010). The roles of tonal and segmental information in Mandarin spoken word recognition: an eyetracking study. *J. Mem. Lang.* 62 407–420. 10.1016/j.jml.2010.02.004

[B33] Malins J. G. Joanisse M. F. (2012a). Setting the tone: an ERP investigation of the influences of phonological similarity on spoken word recognition in Mandarin Chinese. *Neuropsychologia* 50 2032–2043. 10.1016/j.neuropsychologia.2012.05.002 22595659

[B34] Malins J. G. Joanisse M. F. (2012b). “Towards a model of tonal processing during Mandarin spoken word recognition,” in *Proceedings of the Tonal Aspects of Languages-Third International Symposium*, Nanjing.

[B35] Malins J. G. Gao D. Tao R. Booth J. R. Shu H. Joanisse M. F. (2014). Developmental differences in the influence of phonological similarity on spoken word processing in Mandarin Chinese. *Brain Lang.* 138 38–50. 10.1016/j.bandl.2014.09.002 25278419 PMC4252245

[B36] Marslen-Wilson W. D. (1987). Functional parallelism in spoken word-recognition. *Cognition* 25 71–102. 10.1016/0010-0277(87)90005-9 3581730

[B37] McClelland J. L. Elman J. L. (1986). The TRACE model of speech perception. *Cognit. Psychol.* 18 1–86. 3753912 10.1016/0010-0285(86)90015-0

[B38] Miellet S. O’Donnell P. J. Sereno S. C. (2009). Parafoveal magnification: visual acuity does not modulate the perceptual span in reading. *Psychol. Sci.* 20 721–728. 10.1111/j.1467-9280.2009.02364.x 19470124

[B39] Mirman D. (2014). *Growth Curve Analysis and Visualization Using R.* Boca Raton, FL: CRC Press.

[B40] Mirman D. Dixon J. A. Magnuson J. S. (2008). Statistical and computational models of the visual world paradigm: growth curves and individual differences. *J. Mem. Lang.* 59 475–494. 10.1016/j.jml.2007.11.006 19060958 PMC2593828

[B41] Mok P. (2009). On the syllable-timing of Cantonese and Beijing Mandarin. *Chinese J. Phonetics* 2 148–154.

[B42] Norris D. (1994). Shortlist: a connectionist model of continuous speech recognition. *Cognition* 52 189–234. 10.1037/0033-295X.115.2.357 18426294

[B43] O’Seaghdha P. G. Chen J.-Y. Chen T.-M. (2010). Proximate units in word production: phonological encoding begins with syllables in Mandarin Chinese but segments in English. *Cognition* 115 282–302. 10.1016/j.cognition.2010.01.001 20149354 PMC2854551

[B44] Reinisch E. Jesse A. McQueen J. M. (2010). Early use of phonetic information in spoken word recognition: lexical stress drives eye movements immediately. *Q. J. Exp. Psychol.* 4 772–783. 10.1080/17470210903104412 19691004

[B45] Repp B. H. Lin H. (1990). Integration of segmental and tonal information in speech perception: a cross-linguistic study. *J. Phonetics* 18 481–495. 10.1016/s0095-4470(19)30410-3

[B46] Schirmer A. Tang S. Penney T. B. Gunter T. C. Chen H. (2005). Brain responses to segmentally and tonally induced semantic violations in Cantonese. *J. Cogn. Neurosci.* 17 1–12. 10.1162/0898929052880057 15701235

[B47] Sereno J. A. Lee H. (2015). The contribution of segmental and tonal information in Mandarin spoken word processing. *Lang. Speech* 58 131–151. 10.1177/0023830914522956 26677639

[B48] Shattuck-Hufnagel S. Turk A. E. (1996). A prosody tutorial for investigators of auditory sentence processing. *J. Psycholinguist. Res.* 25 193–247. 10.1007/BF01708572 8667297

[B49] Shaw J. A. Tyler M. D. (2020). Effects of vowel coproduction on the timecourse of tone recognition. *J. Acoust. Soc. Am.* 147 2511–2524. 10.1121/10.0001103 32359304

[B50] Shuai L. Malins J. G. (2017). Encoding lexical tones in jTRACE: a simulation of monosyllabic spoken word recognition in Mandarin Chinese. *Behav. Res. Methods* 49 230–241. 10.3758/s13428-015-0690-0 26850055 PMC4975679

[B51] Sun C. C. Hendrix P. Ma J. Q. Baayen R. H. (2018). Chinese Lexical Database (CLD): a large-scale lexical database for simplified Mandarin Chinese. *Behav. Research Methods* 50 2606–2629. 10.3758/s13428-018-1038-3 29934697

[B52] Taft M. Chen H. (1992). “Judging homophony in Chinese: the influence of tones,” in *Language Processing in Chinese*, eds Chen H. Tzeng O. J. L. (Oxford: North-Holland), 151–172. 10.1016/s0166-4115(08)61891-9

[B53] Tanenhaus M. K. Spivey-Knowlton M. J. Eberhard K. M. Sedivy J. C. (1995). Integration of visual and linguistic information in spoken language comprehension. *Science* 268 1632–1634. 7777863 10.1126/science.7777863

[B54] Tong X. McBride C. Burnham D. (2014). Cues for lexical tone perception in children: acoustic correlates and phonetic context effects. *J. Speech Lang. Hear. Res.* 57 1589–1605. 10.1044/2014_JSLHR-S-13-0145 24817506

[B55] Tong Y. Francis A. L. Gandour J. T. (2008). Processing dependencies between segmental and suprasegmental features in Mandarin Chinese. *Lang. Cogn. Process.* 23 689–708.

[B56] Tsai C.-H. (2000). *Mandarin Syllable Frequency Counts for Chinese Characters.* Available online at: http://technology.chtsai.org/syllable/ (accessed April 1, 2000).

[B57] Wiener S. Turnbull R. (2016). Constraints of tones, vowels and consonants on lexical selection in Mandarin Chinese. *Lang. Speech* 59 59–82. 10.1177/0023830915578000 27089806

[B58] Ye Y. Connie C. M. (1999). Processing spoken Chinese: the role of tone information. *Lang. Cogn. Process. Special Issue Process. East Asian Lang.* 14 5–6.

[B59] Yip M. C. (2007). Processing of segmental and tonal information of Chinese syllables. *Percept. Mot. Skills* 105 405–410. 10.2466/pms.105.2.405-410 18065063

[B60] You W. Zhang Q. Verdonschot R. G. (2012). Masked syllable priming effects in words and picture naming in Chinese. *PLoS One* 7:e46595. 10.1371/journal.pone.0046595 PMC3466322 23056360

[B61] Zhang X. Samuel A. G. Liu S. (2012). The perception and representation of segmental and prosodic Mandarin contrasts in native speakers of Cantonese. *J. Mem. Lang.* 66 438–457. 10.1016/j.jml.2011.12.006 22707849 PMC3374417

[B62] Zhao J. Guo J. Zhou F. Shu H. (2011). Time course of Chinese monosyllabic spoken word recognition: evidence from ERP analyses. *Neuropsychologia* 49 1761–1770. 10.1016/j.neuropsychologia.2011.02.054 21382389

[B63] Zhou X. Marslen-Wilson W. (1994). Words, morphemes, and syllables in the Chinese mental lexicon. *Lang. Cogn. Process.* 9 393–422.

[B64] Ziegler J. C. Ferrand L. (1998). Orthography shapes the perception of speech: the consistency effect in auditory word recognition. *Psychon. Bull. Rev.* 5 683–689. 10.1016/j.cognition.2006.12.005 17250820

[B65] Zou L. Chen Y. (2019). “The roles of tonal and segmental information in spoken word recognition for L2 speakers: evidence from dutch learners of mandarin,” in *Proceedings of the 19th International Congress of Phonetic Sciences*, Melbourne, VIC.

[B66] Zou L. Desroches A. S. Liu Y. Xia Z. Shu H. (2012). Orthographic facilitation in Chinese spoken word recognition: an ERP study. *Brain Lang.* 123 164–173. 10.1016/j.bandl.2012.09.006 23098916

[B67] Zou T. (2017). *Production and Perception of Tones by Dutch Learners of Mandarin.* Utrecht: LOT.

