# Supplementary material for: The Roles of Consonant, Rime, and Tone in Mandarin Spoken Word Recognition: An Eye-Tracking Study
Source: Front Psychol. 2022 Jan 5;12:740444. doi: 10.3389/fpsyg.2021.740444 (PMC8766742; doi:10.3389/fpsyg.2021.740444)
Supplement: Supplementary file 1 [file Data_Sheet_1.pdf]

Appendix

Pictures used for target and competitor conditions. For each stimulus, the written form, corresponding picture, pinyin, English translation, average rating point for word-picture matching, and times of incorrect naming in the training section are presented. Tone pair in the table refers to the tone contrasts between the target words and Consonant+Rime competitors.

Tone pair	Target	Consonant	Rime	Tone	Consonant+Rime	Consonant+Tone	Rime+Tone	Cohort	Cohort+Tone	Baseline
T1-T2	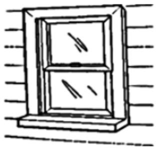<br>**窗 chuang1**<br>**(window)**<br>rating: 6.75<br>incorrect: 0	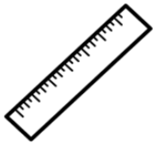<br>**尺 chi3**<br>**(ruler)**<br>rating: 7<br>incorrect: 0	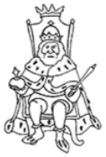<br>**王 wang2**<br>**(king)**<br>rating: 7<br>incorrect: 0	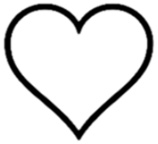<br>**心 xin1**<br>**(heart)**<br>rating: 6.88<br>incorrect: 0	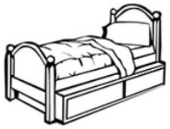<br>**床 chuang2**<br>**(bed)**<br>rating: 7<br>incorrect: 0	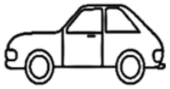<br>**车 che1**<br>**(car)**<br>rating: 7<br>incorrect: 0	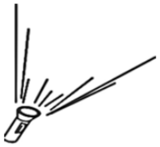<br>**光 guang1**<br>**(light)**<br>rating: 6.5<br>incorrect: 5	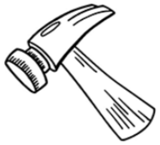<br>**锤 chui2**<br>**(hammer)**<br>rating: 6.75<br>incorrect: 2	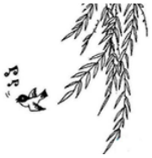<br>**春 chun1**<br>**(spring)**<br>rating: 6.38<br>incorrect: 8	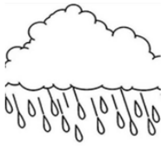<br>**雨 yu3**<br>**(rain)**<br>rating: 6.83<br>incorrect: 0
T1-T3	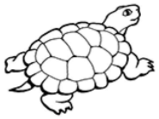<br>**龟 gui1**<br>**(turtle)**<br>rating: 7<br>incorrect: 0	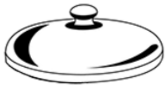<br>**盖 gai4**<br>**(lid)**<br>rating: 6.75<br>incorrect: 0	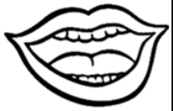<br>**嘴 zui3**<br>**(mouth)**<br>rating: 6.83<br>incorrect: 0	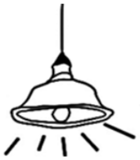<br>**灯 deng1**<br>**(lamp)**<br>rating: 7<br>incorrect: 0	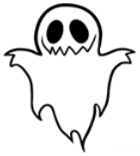<br>**鬼 gui3**<br>**(ghost)**<br>rating: 6.75<br>incorrect: 0	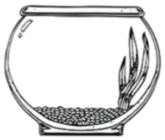<br>**缸 gang1**<br>**(jar)**<br>rating: 6.38<br>incorrect: 1	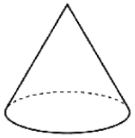<br>**锥 zhui1**<br>**(cone)**<br>rating: 6.75<br>incorrect: 0	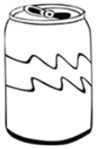<br>**罐 guan4**<br>**(can)**<br>rating: 6.88<br>incorrect: 3	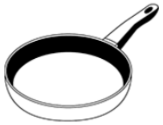<br>**锅 guo1**<br>**(pan)**<br>rating: 7<br>incorrect: 4	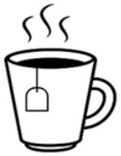<br>**茶 cha2**<br>**(tea)**<br>rating: 7<br>incorrect: 3

Tone pair	Target	Consonant	Rime	Tone	Consonant+Rime	Consonant+Tone	Rime+Tone	Cohort	Cohort+Tone	Baseline
T1-T4	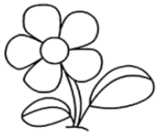<br>**花 hua1**<br>**(flower)**<br>rating: 7<br>incorrect: 0	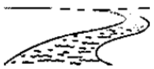<br>**河 he2**<br>**(river)**<br>rating: 6.88<br>incorrect: 5	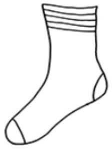<br>**袜 wa4**<br>**(sock)**<br>rating: 7<br>incorrect: 0	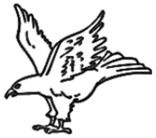<br>**鹰 ying1**<br>**(eagle)**<br>rating: 6.88<br>incorrect: 0	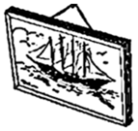<br>**画 hua4**<br>**(painting)**<br>rating: 6.38<br>incorrect: 0	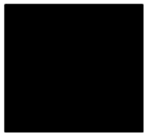<br>**黑 hei1**<br>**(black)**<br>rating: 6.75<br>incorrect: 0	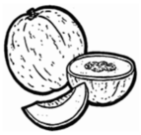<br>**瓜 gua1**<br>**(melon)**<br>rating: 6.75<br>incorrect: 1	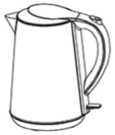<br>**壶 hu2**<br>**(kettle)**<br>rating: 7<br>incorrect: 1	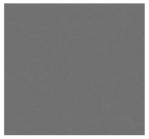<br>**灰 hui1**<br>**(grey)**<br>rating: 6.38<br>incorrect: 0	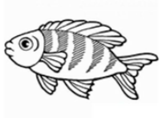<br>**鱼 yu2**<br>**(fish)**<br>rating: 7<br>incorrect: 0
T2-T1	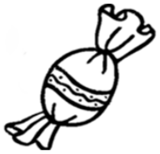<br>**糖 tang2**<br>**(candy)**<br>rating: 6.88<br>incorrect: 0	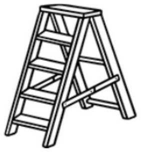<br>**梯 ti1**<br>**(ladder)**<br>rating: 7<br>incorrect: 1	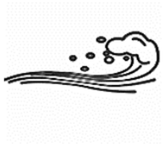<br>**浪 lang4**<br>**(wave)**<br>rating: 6.38<br>incorrect: 4	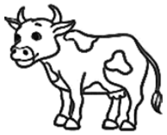<br>**牛 niu2**<br>**(cow)**<br>rating: 7<br>incorrect: 0	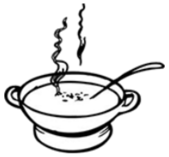<br>**汤 tang1**<br>**(soup)**<br>rating: 6.63<br>incorrect: 1	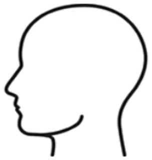<br>**头 tou2**<br>**(head)**<br>rating: 6.75<br>incorrect: 4	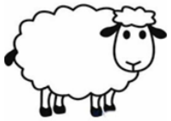<br>**羊 yang2**<br>**(sheep)**<br>rating: 7<br>incorrect: 0	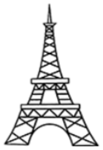<br>**塔 ta3**<br>**(tower)**<br>rating: 7<br>incorrect: 0	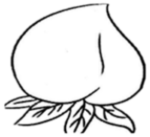<br>**桃 tao2**<br>**(peach)**<br>rating: 7<br>incorrect: 0	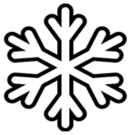<br>**雪 xue3**<br>**(snow)**<br>rating: 6.88<br>incorrect: 0

Tone pair	Target	Consonant	Rime	Tone	Consonant+Rime	Consonant+Tone	Rime+Tone	Cohort	Cohort+Tone	Baseline
T2-T3	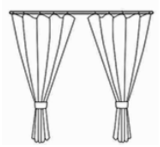<br>**帘 lian2**<br>**(curtain)**<br>rating: 6.88<br>incorrect: 0	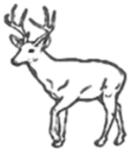<br>**鹿 lu4**<br>**(deer)**<br>rating: 7<br>incorrect: 1	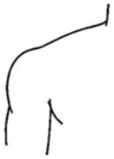<br>**肩 jian1**<br>**(shoulder)**<br>rating: 6.13<br>incorrect: 1	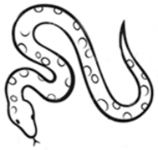<br>**蛇 she2**<br>**(snake)**<br>rating: 7<br>incorrect: 0	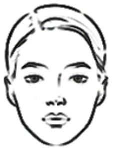<br>**脸 lian3**<br>**(face)**<br>rating: 6.75<br>incorrect: 2	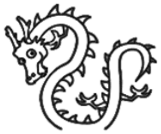<br>**龙 long2**<br>**(dragon)**<br>rating: 7<br>incorrect: 0	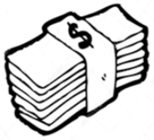<br>**钱 qian2**<br>**(money)**<br>rating: 7<br>incorrect: 0	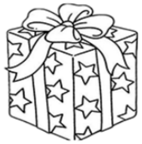<br>**礼 li3**<br>**(gift)**<br>rating: 6.38<br>incorrect: 2	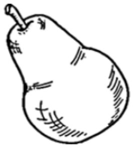<br>**梨 li2**<br>**(pear)**<br>rating: 6.63<br>incorrect: 0	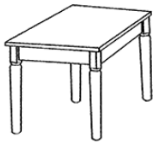<br>**桌 zhuo1**<br>**(desk)**<br>rating: 7<br>incorrect: 0
T2-T4	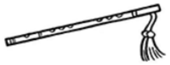<br>**笛 di2**<br>**(flute)**<br>rating: 6.75<br>incorrect: 1	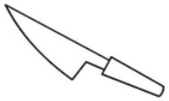<br>**刀 dao1**<br>**(knife)**<br>rating: 7<br>incorrect: 0	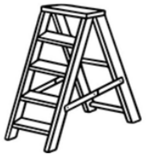<br>**梯 ti1**<br>**(ladder)**<br>rating: 7<br>incorrect: 1	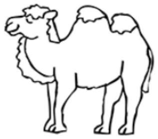<br>**驼 tuo2**<br>**(camel)**<br>rating: 7<br>incorrect: 1	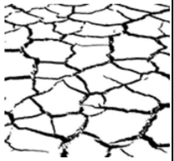<br>**地 di4**<br>**(land)**<br>rating: 6.38<br>incorrect: 6	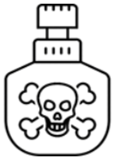<br>**毒 du2**<br>**(poison)**<br>rating: 6.88<br>incorrect: 0	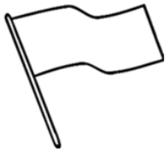<br>**旗 qi2**<br>**(flag)**<br>rating: 7<br>incorrect: 0	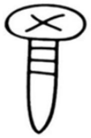<br>**钉 ding1**<br>**(nail)**<br>rating: 6.88<br>incorrect: 0	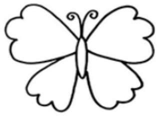<br>**蝶 die2**<br>**(butterfly)**<br>rating: 7<br>incorrect: 1	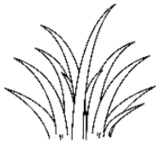<br>**草 cao3**<br>**(grass)**<br>rating: 6.88<br>incorrect: 1

Tone pair	Target	Consonant	Rime	Tone	Consonant+Rime	Consonant+Tone	Rime+Tone	Cohort	Cohort+Tone	Baseline
T3-T1	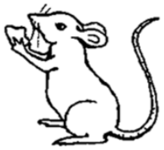<br>**鼠 shu3**<br>**(mouse)**<br>rating: 7<br>incorrect: 1	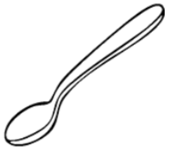<br>**勺 shao2**<br>**(spoon)**<br>rating: 6.88<br>incorrect: 0	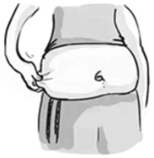<br>**肚 du4**<br>**(tummy)**<br>rating: 6.75<br>incorrect: 0	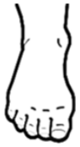<br>**脚 jiao3**<br>**(foot)**<br>rating: 7<br>incorrect: 1	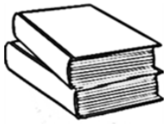<br>**书 shu1**<br>**(book)**<br>rating: 6.75<br>incorrect: 0	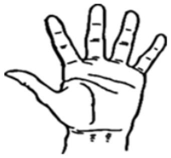<br>**手 shou3**<br>**(hand)**<br>rating: 6.88<br>incorrect: 1	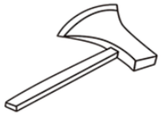<br>**斧 fu3**<br>**(axe)**<br>rating: 7<br>incorrect: 0	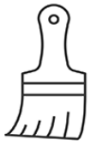<br>**刷 shua1**<br>**(brush)**<br>rating: 6.88<br>incorrect: 0	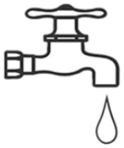<br>**水 shui3**<br>**(water)**<br>rating: 6.38<br>incorrect: 1	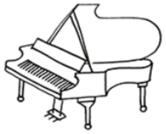<br>**琴 qin2**<br>**(piano)**<br>rating: 6.88<br>incorrect: 1
T3-T2	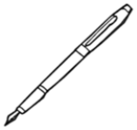<br>**笔 bi3**<br>**(pen)**<br>rating: 6.88<br>incorrect: 0	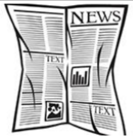<br>**报 bao4**<br>**(newspaper)**<br>rating: 6.88<br>incorrect: 0	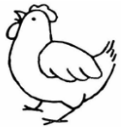<br>**鸡 ji1**<br>**(chicken)**<br>rating: 6.88<br>incorrect: 1	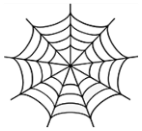<br>**网 wang3**<br>**(net)**<br>rating: 7<br>incorrect: 0	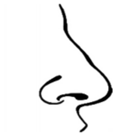<br>**鼻 bi2**<br>**(nose)**<br>rating: 7<br>incorrect: 0	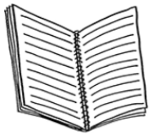<br>**本 ben3**<br>**(notebook)**<br>rating: 6.5<br>incorrect: 8	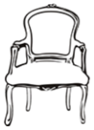<br>**椅 yi3**<br>**(chair)**<br>rating: 7<br>incorrect: 0	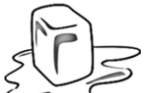<br>**冰 bing1**<br>**(ice)**<br>rating: 5.63<br>incorrect: 0	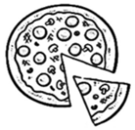<br>**饼 bing3**<br>**(pie)**<br>rating: 6.13<br>incorrect: 1	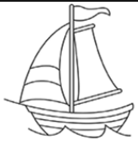<br>**船 chuan2**<br>**(boat)**<br>rating: 6.88<br>incorrect: 1

Tone pair	Target	Consonant	Rime	Tone	Consonant+Rime	Consonant+Tone	Rime+Tone	Cohort	Cohort+Tone	Baseline
T3-T4	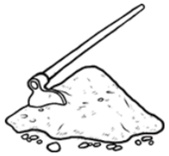<br>**土 tu3**<br>**(dirt)**<br>rating: 5.5<br>incorrect: 2	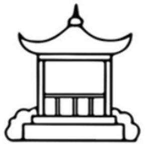<br>**亭 ting2**<br>**(pavilion)**<br>rating: 7<br>incorrect: 0	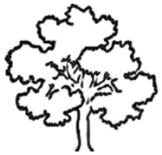<br>**树 shu4**<br>**(tree)**<br>rating: 7<br>incorrect: 0	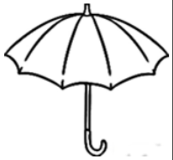<br>**伞 san3**<br>**(umbrella)**<br>rating: 7<br>incorrect: 0	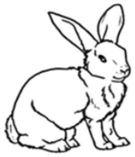<br>**兔 tu4**<br>**(rabbit)**<br>rating: 6.88<br>incorrect: 0	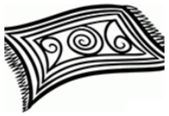<br>**毯 tan3**<br>**(rug)**<br>rating: 6.5<br>incorrect: 0	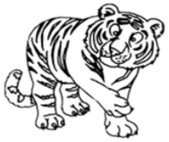<br>**虎 hu3**<br>**(tiger)**<br>rating: 6.88<br>incorrect: 0	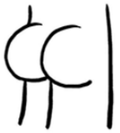<br>**臀 tun2**<br>**(hip)**<br>rating: 6.5<br>incorrect: 0	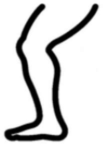<br>**腿 tui3**<br>**(leg)**<br>rating: 7<br>incorrect: 1	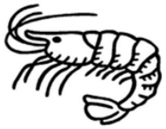<br>**虾 xia1**<br>**(shrimp)**<br>rating: 6.88<br>incorrect: 0
T4-T1	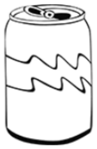<br>**罐 guan4**<br>**(can)**<br>rating: 6.88<br>incorrect: 3	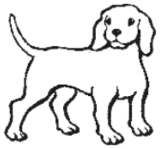<br>**狗 gou3**<br>**(dog)**<br>rating: 6.75<br>incorrect: 0	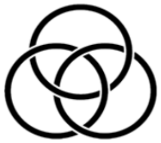<br>**环 huan2**<br>**(ring)**<br>rating: 7<br>incorrect: 0	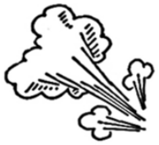<br>**气 qi4**<br>**(gas)**<br>rating: 5.5<br>incorrect: 1	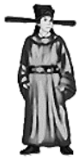<br>**官 guan1**<br>**(officer)**<br>rating: 6.88<br>incorrect: 0	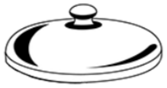<br>**盖 gai4**<br>**(lid)**<br>rating: 6.75<br>incorrect: 0	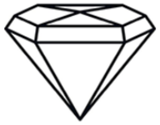<br>**钻 zuan4**<br>**(diamond)**<br>rating: 6.88<br>incorrect: 1	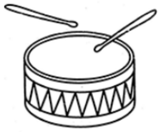<br>**鼓 gu3**<br>**(drum)**<br>rating: 7<br>incorrect: 1	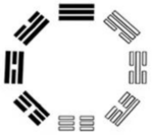<br>**卦 gua4**<br>**(Bagua)**<br>rating: 6.75<br>incorrect: 1	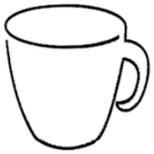<br>**杯 bei1**<br>**(cup)**<br>rating: 7<br>incorrect: 0
